# Applications of extracellular vesicles in tissue regeneration: a narrative review

**DOI:** 10.3389/fbioe.2026.1876356

**Published:** 2026-08-24

**Authors:** Nabin Rokaya, Krishna Prasad Shetty, Vijay Desai, Dinesh Rokaya

**Affiliations:** 1 Department of Dermatology, Kathmandu Clinic of Cosmetic Surgery (KCCS), Kathmandu, Nepal; 2 Clinical Sciences Department, College of Dentistry, Ajman University, Ajman, United Arab Emirates; 3 Center of Medical and Bio-Allied Health Sciences Research, Ajman University, Ajman, United Arab Emirates

**Keywords:** dentistry, exosomes, extracellular vesicles, medicine, regeneration

## Abstract

Extracellular vesicles (EVs) are nanoscale, membrane-bound particles released by most cell types and play essential roles in intercellular communication. By transporting proteins, lipids, and nucleic acids, they regulate inflammation, angiogenesis, immune responses, and tissue regeneration. Their biocompatibility and ability to deliver targeted molecular cargo make them promising diagnostic and therapeutic tools. In dentistry, EVs support periodontal repair, dentin–pulp regeneration, bone healing, and implant integration. They are also valuable as salivary biomarkers for oral and systemic diseases. Beyond dentistry, EVs are being explored in oncology, cardiovascular repair, neurology, and regenerative medicine as versatile cell-free therapeutic platforms. This review provides a comprehensive overview of the biology, biogenesis, physiological functions, and mechanisms of action of extracellular vesicles, with a particular focus on their emerging applications in tissue regeneration. A literature search was conducted using PubMed and Google Scholar databases for studies published between 2010 and 2025. Original research articles, review papers, and relevant book chapters published in English were included. EVs have emerged as powerful cell-free mediators of regeneration, capable of orchestrating osteogenesis, angiogenesis, immunomodulation, and tissue repair across maxillofacial structures. Their diverse cargo of miRNAs, proteins, and growth factors enables precise modulation of cellular signaling pathways essential for healing. Current evidence highlights their promising applications in periodontal, pulp–dentin, and craniofacial bone regeneration, as well as their diagnostic value through salivary biomarkers. Continued advancements in EVs standardization, large-scale production, and clinical translation will be critical for integrating EVs-based therapies into future regenerative dentistry and maxillofacial medicine.

## 1 Introduction

Extracellular vesicles (EVs) are bilayer lipid-bound vesicles with a size ranging from approximately 30 nm to above 1 μm and are released by cells into the extracellular space to transport proteins, lipids, and nucleic acids for cell-to-cell communication (Ginini et al., 2022; Doyle and Wang, 2019). EVs are secreted by nearly all cell types and serve as important mediators of intercellular communication. These vesicles carry a diverse cargo of bioactive molecules, including proteins, lipids, messenger RNAs, microRNAs, and other nucleic acids, which can influence the behavior and function of recipient cells (Amin et al., 2024; Berumen Sánchez et al., 2021). Through the transfer of these molecular signals, EVs participate in numerous physiological and pathological processes, including tissue homeostasis, immune regulation, angiogenesis, inflammation, and regeneration (Sun et al., 2024a; Wang and Pan, 2023).

There are 3 basic subtypes of EVs based upon content, size, function, and biogenesis: exosomes, microvesicles, and apoptotic bodies (Doyle and Wang, 2019; Rajput et al., 2022). Exosomes act as natural nano-sized membrane particles, containing proteins, lipids, and nucleic acids, and they have the potential for the rejuvenation of epithelial and mesenchymal tissues. (Xing et al., 2020; Hessvik et al., 2016; Donoso Quezada et al., 2021). Apoptotic bodies are approximately 500–2000 nm, and they are released into the circulation during the programmed and coordinated cell death in the body (Santavanond et al., 2021). Finally, microvesicles are approximately 100–1,000 nm in size and are formed directly by budding from the plasma membrane (Carvalho Ferraz et al., 2025; Schank et al., 2026). The multivesicular bodies (MVBs) are formed through both endosomal sorting complex required for transport (ESCRT)-dependent and ESCRT-independent pathways. In the ESCRT-dependent pathway, ESCRT-0, ESCRT-I, ESCRT-II, and ESCRT-III complexes coordinate cargo sorting, membrane invagination, and vesicle scission (Wollert and Hurley, 2010). They are formed intracellularly through inward budding of the limiting membrane of endocytic compartments, forming vesicle-containing endosomes called MVBs (Doyle and Wang, 2019). ESCRT-independent mechanisms involve lipid-mediated processes, particularly ceramide generation by neutral sphingomyelinase, as well as tetraspanins such as CD9, CD63, and CD81, which facilitate membrane organization and internal vesicle formation (van Niel et al., 2018). Following maturation, MVBs either fuse with lysosomes for degradation or merge with the plasma membrane to release internal vesicles as exosomes into the extracellular environment (blood, urine, saliva, CSF, etc.) and culture media (Rajput et al., 2022; Krylova and Feng, 2023).

The peculiar cellular signaling potential of the EVs has justified the modification of cellular function, which has revolutionized the treatment modalities in the field of regenerative medicine. A literature search was conducted using PubMed and Google Scholar databases for studies published between 2010 and 2025. Original research articles, review papers, and relevant book chapters published in English were included.

## 2 Source and production of extracellular vesicles

**FIGURE 1:**
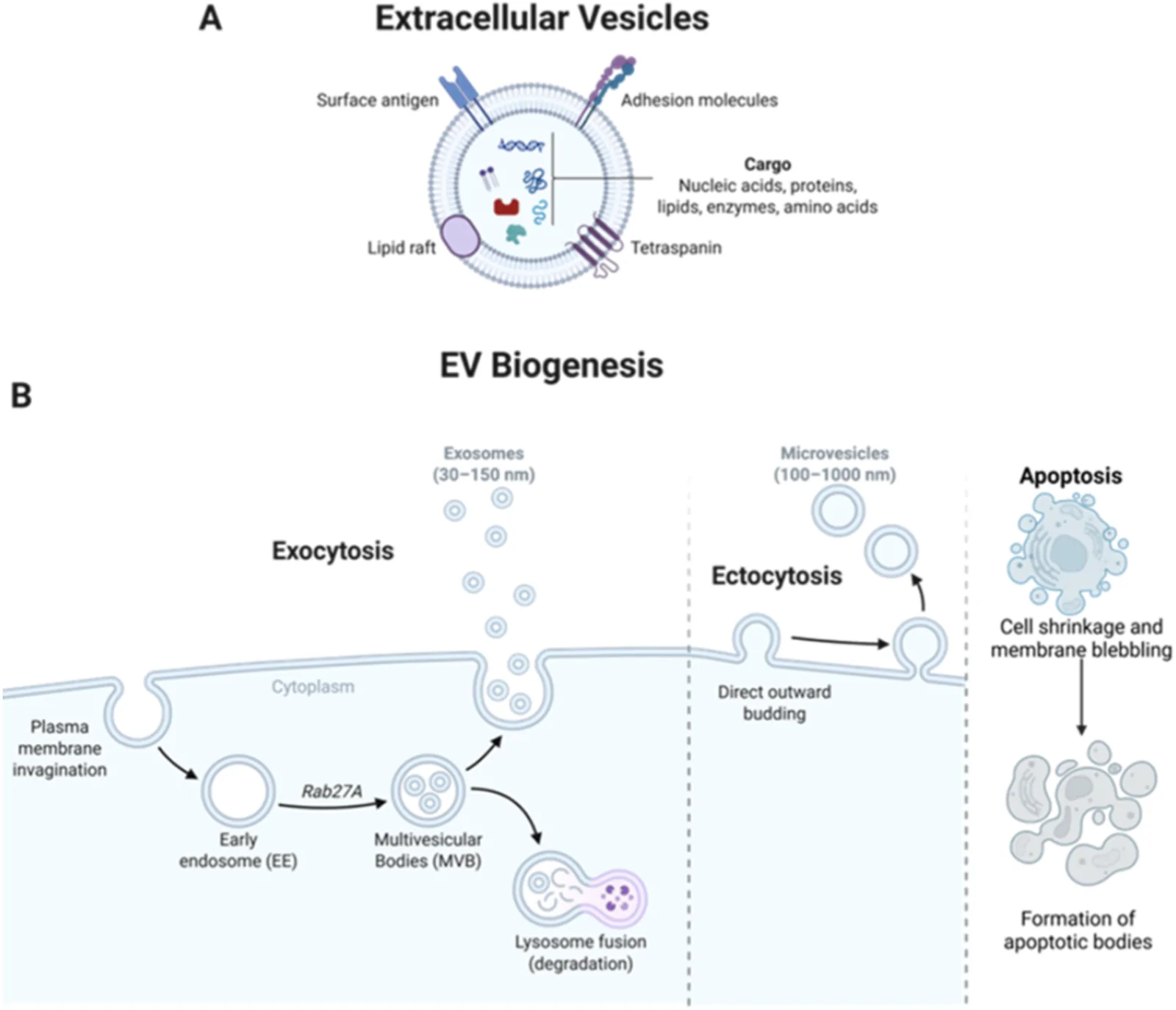
Extracellular vesicles (EVs) structure and biogenesis pathways. **(A)** EVs contain tetraspanin proteins and encapsulate amino acids, RNA, DNA, proteins, and enzymes that allow for essential intercellular communication. **(B)** Exosomes are generated through the endosomal pathway, where multivesicular bodies release intraluminal vesicles into the extracellular space via exocytosis, enabling intercellular communication. Adapted with permission from Ref (Schank et al., 2026). under Creative Commons Attribution (CC BY) license.

Production and secretion of EVs are heavily influenced by age, gender, disease, fasting state, medication, etc. There is an optimal production of these nanoparticles under ideal environmental conditions for cellular homeostasis (Salomon et al., 2022; Li M. et al., 2022). The different types of EVs are produced in different ways, either forming endosomal bodies, forming blebs on the outer membrane, or being released from dead cells. Figure 1 shows the EVs structure and biogenesis pathways (Schank et al., 2026). EVs contain tetraspanin proteins and encapsulate amino acids, RNA, DNA, proteins, and enzymes for intercellular communication (Petroni et al., 2023).

The exosomes are generated through the endosomal pathway, where multivesicular bodies release intraluminal vesicles into the extracellular space via exocytosis, enabling intercellular communication (Gurung et al., 2021; Xu et al., 2023). In contrast, microvesicles are produced by direct outward budding of the plasma membrane, whereas apoptotic bodies are released during programmed cell death through membrane blebbing (Ståhl et al., 2019; Gregory and Rimmer, 2023).

Finally, EVs get separated from the parental cells and are released outside the cell (Jing et al., 2018). Once they are detached from the original cells into the extracellular compartment, they are transported to distant target cells/tissues by bodily fluids for cellular communication. These EVs have important biological roles as they have transformed the functions of different cells in regenerative medicine (Petroni et al., 2023; Yáñez-Mó et al., 2015). These cell-derived nanoparticles are often loaded with genetic material, such as proteins and lipids, which could play a role in the intracellular signaling pathway for cellular processes (Kim et al., 2024). All cells in human secrete different EVs in different forms throughout their lifespan, either during normal physiological processes or at the time of ongoing pathological processes. They play a crucial role in cancer prognosis and diagnosis, as they are secreted by both healthy and tumor cells (Zhang et al., 2012). This can reduce the invasive procedures for the oral cancer diagnosis.

**FIGURE 2:**
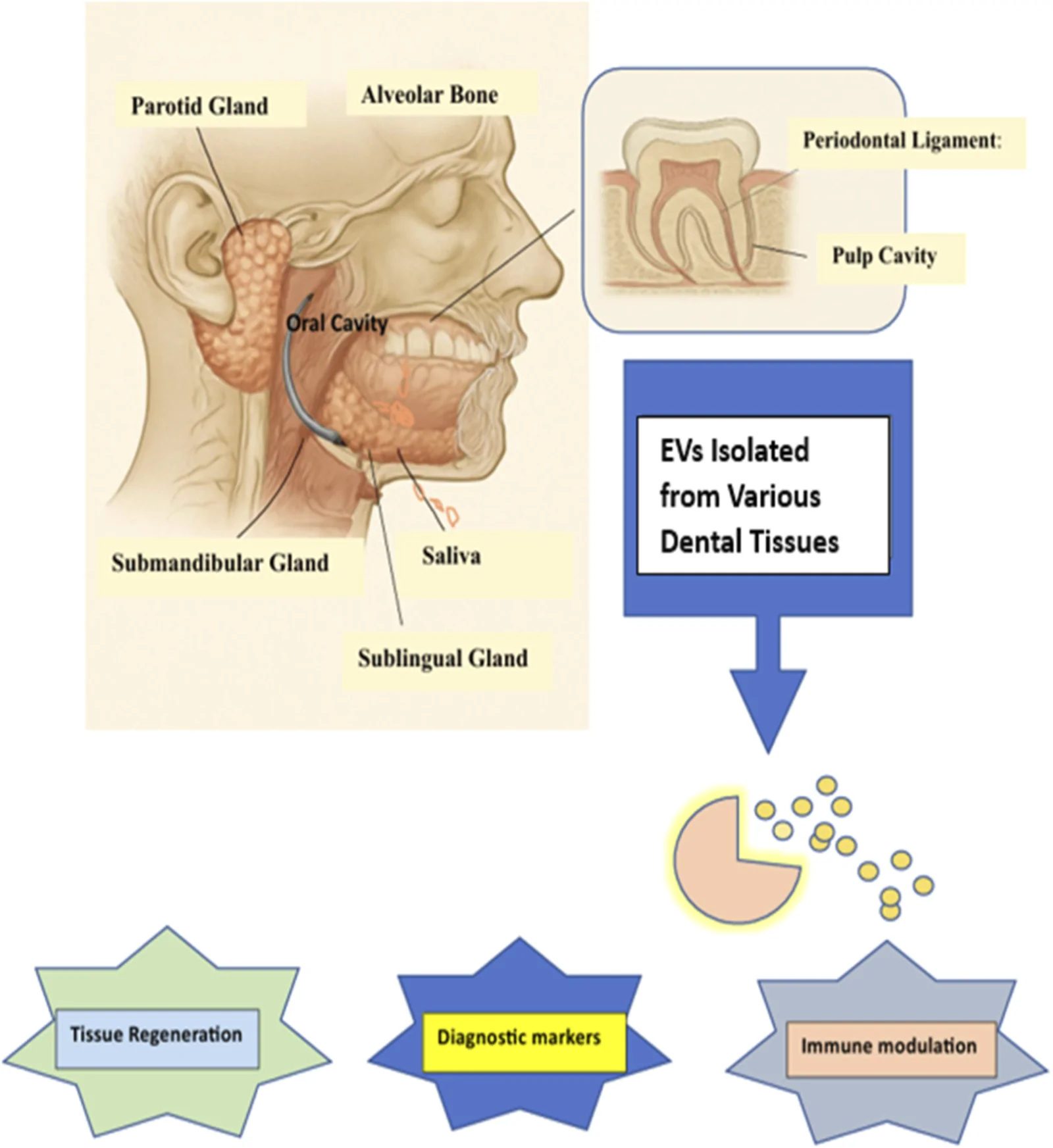
Sources and origins of extracellular vehicles (EVs). EVs can be isolated from various dental tissues within the oral cavity, such as the periodontal ligament, alveolar bone, gingiva, and salivary glands. These EVs play a pivotal role in modulating the regeneration of damaged dental tissues and contribute significantly to diagnostic applications and immune modulation. Figure created on Microsoft PowerPoint 2021 (Microsoft Corp., Redmond, Washington, United States).

The EVs are typically secreted by almost every cell and act as mediators of intercellular communication. As a result of this, these nanoparticles can be extracted from various tissue sites in the dental field, each providing a unique potential for regenerative therapies, as shown in Figure 2. The major sources of cell-derived EVs in dental tissues are described in the following sections. Table 1 presents a summary of sources of EVs in the oral cavity and their characteristics.

**TABLE 1:** Summary of major sources of EVs in the oral cavity and their characteristics.

Oral tissues	Cells of origin	Major EV cargo	Characteristics
Dental pulp	Dental pulp stem cells (DPSCs), odontoblasts	miRNAs, dentin matrix proteins, VEGF, BMP-2, TGF-β	Promote odontogenic differentiation, angiogenesis, neurogenesis, anti-inflammatory activity, pulp regeneration
Periodontal ligament (PDL)	Periodontal ligament stem cells (PDLSCs), fibroblasts	miRNAs (miR-21, miR-17-5p, miR-155), growth factors, cytokines, proteins	Promote osteogenic differentiation, periodontal regeneration, angiogenesis, immunomodulation, inhibit inflammation
Dental follicle	Dental follicle stem cells (DFSCs)	Osteogenic proteins, Wnt-related molecules, miRNAs	Support osteogenesis, periodontal tissue formation, immunomodulation
Alveolar bone	Bone marrow mesenchymal stem cells (BMSCs), osteoblasts, osteocytes	Osteogenic miRNAs (miR-196a, miR-27a), BMPs, VEGF, ALP, RUNX2-related proteins	Stimulate osteogenesis, mineralization, angiogenesis, bone remodeling, regulate osteoclast activity
Salivary glands	Acinar cells, ductal epithelial cells, salivary gland stem/progenitor cells	Salivary proteins, antimicrobial peptides, miRNAs, mRNAs, enzymes	Maintain oral homeostasis, antimicrobial defense, immune regulation, epithelial repair, facilitate cell-to-cell communication

### 2.1 Dental pulp

Dental pulp stem cells (DPSCs), residing within the dental pulp cavity, possess remarkable regenerative and reparative potential (Chansaenroj et al., 2025). EVs derived from DPSCs serve as important mediators of intercellular communication by transporting a variety of bioactive molecules, including growth factors, proteins, lipids, and nucleic acids (Sun et al., 2024a; Lorite et al., 2024). These vesicles promote tissue repair and regeneration through signaling pathways involving collagen synthesis, vascular endothelial growth factor (VEGF), fibroblast growth factor (FGF), and other regenerative mediators (Zhang et al., 2024). EVs released by DPSCs recruit endogenous progenitor cells and modulate the local microenvironment to favor tissue healing. Consequently, DPSC-derived EVs contribute to dentin regeneration, pulp tissue repair, and restoration of the pulp–dentin complex, highlighting their promising therapeutic potential in regenerative endodontics (Vafaei et al., 2022; Matte et al., 2023).

### 2.2 Periodontal ligament

Another important site where EVs can be extracted is the periodontal Ligament. These vesicles, derived from the periodontal ligament stem cells (PDLSCs), bear periodontal tissue regenerating capacities that include the restoration of the diseased periodontal ligament, alveolar bone, and cementum (Sh et al., 2025; Ahmad et al., 2025). EVs taken from PDL can express the signaling involving the production of FGF that promotes regeneration of PDL cells and helps in the stimulation of dental pulp stem cells (Hua et al., 2021).

### 2.3 Dental follicle

Dental follicle, one of the mesenchymal tissues, plays a significant role in cellular communication for the formation of PDL, alveolar bone, and other dental tissues (Zhou et al., 2019). EVs derived from dental follicle cells (DFCs) are found to possess proteins such as collagen, cytokines, BMP, VEGF, etc., which modulate odontogenesis (Sun et al., 2024b; Zou et al., 2023). The miRNA and transforming growth factor-beta (TGF-β), signaled by follicular exosomes, a subclass of EVs, regulate the inflammatory cascade and stimulate the tissue healing process in dental tissue such as PDL, cementum, and alveolar bone (Bi et al., 2021).

### 2.4 Alveolar bone

Osteoblasts can express EVs that can be utilized in the bone renewal process. Bone morphogenetic proteins (BMPs), such as BMP-2 and BMP-7, play an integral part in osteogenesis, thereby stimulating bone growth (de Souza et al., 2024). In addition, the exosomes, a subclass of EVs derived from alveolar bone, might possess such proteins, including angiogenic factors like VEGF and other growth factors like FGF and TGF-β, which help in osteoblast differentiation (Biswas et al., 2025). These extracellular vesicles from osteoblasts in the alveolar bone may contribute to bone regeneration, thus providing a ray of hope for the regeneration of bone defects in oral and maxillofacial bony components and eventually supporting the integration of dental implants and prostheses (Lange et al., 2024; Li et al., 2024).

### 2.5 Salivary glands

The secretion of the different salivary glands consists of EVs having a special communication signal released from the oral cavity tissues (Cui et al., 2024; Han et al., 2018). Even though it’s not directly associated with dental tissue, EVs derived from the salivary glands can aid in tissue repair, inflammation reduction, and cellular signaling in the oral cavity, identification of biomarkers of different oral-dental diseases, including cancer, with potential applications in oral health (Sanesi et al., 2024; Yu M. Y. et al., 2024).

Although EVs derived from dental pulp, periodontal ligament, dental follicle, alveolar bone, and salivary glands demonstrate considerable regenerative potential, the current evidence is predominantly based on *in vitro* experiments and animal models, with limited validation in human clinical studies. The regenerative capacity of EVs varies according to their cellular origin, donor characteristics, isolation methods, and cargo composition, making direct comparisons across studies challenging. The precise mechanisms underlying tissue-specific regenerative effects also remain incompletely understood, and standardized protocols for EV isolation, characterization, dosing, and delivery are still lacking. Addressing these limitations through robust mechanistic studies and well-designed clinical trials will be essential for translating tissue-derived EVs into reliable therapeutic strategies for oral and maxillofacial regeneration.

## 3 Signaling pathways of extracellular vesicles

The physiological function of EVs relies on their ability to transfer molecular information between cells. Following release, EVs interact with target cells through receptor–ligand interactions, endocytosis, phagocytosis, or direct membrane fusion, thereby delivering their cargo into the cytoplasm of recipient cells (Yáñez-Mó et al., 2015). Among these, the phosphoinositide 3-kinase/protein kinase B (PI3K/Akt) pathway plays a crucial role in promoting cell survival and tissue regeneration. EVs-associated miRNAs such as miR-21 and miR-126, together with angiogenic factors such as VEGF, stimulate Akt phosphorylation, leading to enhanced cellular proliferation and reduced apoptosis (Mathieu et al., 2019).

Similarly, EVs modulate the mitogen-activated protein kinase/extracellular signal-regulated kinase (MAPK/ERK) pathway, which regulates cell growth and wound healing. The Wnt/β-catenin signaling pathway, activated by EVs-associated Wnt proteins and regulatory miRNAs, promotes stem cell differentiation, osteogenesis, and tissue regeneration. EVs influence extracellular matrix remodeling through activation of the TGF-β)/Smad pathway, stimulating collagen synthesis and tissue repair. In addition, EVs-derived anti-inflammatory miRNAs (miR-146a and miR-223) suppress nuclear factor kappa B (NF-κB) signaling, thereby reducing the production of pro-inflammatory cytokines and facilitating tissue healing (Kalluri and LeBleu, 2020).

### 3.1 Signaling pathways involved in EVs-Mediated tissue regeneration

EVs, particularly derived from mesenchymal stem cells (MSCs), DPSCs, PDLSCs, and stem cells from human exfoliated deciduous teeth (SHEDs), promote regeneration of tissues through activation of multiple intracellular signaling pathways (Xia et al., 2024; Mottaghi et al., 2025; Ning et al., 2024). These pathways can regulate osteogenesis, angiogenesis, neurogenesis, chondrogenesis, and immunomodulation, which are essential for successful tissue repair. EVs carry a diverse cargo of bioactive molecules, including proteins, lipids, messenger RNAs (mRNAs), microRNAs (miRNAs), long non-coding RNAs, cytokines, and growth factors, which can influence the behavior and function of recipient cells (Raposo and Stoorvogel, 2013).

One of the most extensively studied pathways is the PI3K/Akt/mTOR pathway, which enhances cellular proliferation, migration, survival, and angiogenesis. EVs-derived cargo activates Akt phosphorylation, leading to increased osteoblastic differentiation and vascularization in bone defects (Liu et al., 2022). However, the evidence is largely derived from *in vitro* studies and small animal models, limiting the direct translation of these findings to clinical practice. Furthermore, the specific EV cargo responsible for PI3K/Akt/mTOR activation remains incompletely characterized, and activation of this pathway may vary depending on the EV source, isolation method, recipient cell type, and local microenvironment. Sustained or excessive PI3K/Akt/mTOR signaling may also have undesirable effects, including dysregulated cell proliferation or fibrosis, highlighting the need for careful modulation of pathway activation.

The Wnt/β-catenin pathway is another key regulator of bone regeneration, stimulating osteogenic differentiation of mesenchymal stem cells and promoting expression of osteogenic markers such as RUNX2 and osteocalcin (Houschyar et al., 2018). EVs-mediated activation of Wnt signaling has been associated with enhanced jawbone and periodontal regeneration. However, current evidence is largely limited to preclinical studies, and its translation to predictable clinical outcomes remains uncertain. In addition, the mechanisms by which EV cargo regulates Wnt/β-catenin signaling are not fully understood, while variability in EV sources and isolation methods contributes to inconsistent findings. Therefore, standardized protocols and robust clinical studies are required to validate the safety and therapeutic efficacy of EV-mediated Wnt signaling in bone regeneration.

The BMP/Smad signaling pathway contributes significantly to osteogenesis by inducing differentiation of progenitor cells into osteoblasts (Biswas et al., 2025). EVs can deliver osteoinductive molecules that enhance BMP signaling, resulting in increased mineral deposition and bone formation. Likewise, the TGF-β/Smad pathway regulates extracellular matrix synthesis, tissue remodeling, and periodontal regeneration (Liao et al., 2026).

For vascularized tissue regeneration, EVs activate the HIF-1α/VEGF pathway, promoting endothelial cell migration and neovascularization. In inflammatory environments, EVs modulate the NF-κB pathway, suppressing pro-inflammatory cytokine production while encouraging macrophage polarization toward the anti-inflammatory M2 phenotype (Palazon et al., 2014). This immunomodulatory effect is crucial for periodontal, pulp, and soft tissue healing.

Additional pathways implicated in maxillofacial regeneration include MAPK/ERK, JAK/STAT, Notch, Hippo/YAP, AMPK, and RANK/RANKL/OPG signaling (Liu et al., 2022). Together, these interconnected pathways coordinate cellular communication, matrix remodeling, angiogenesis, and tissue-specific differentiation, highlighting the multifaceted regenerative potential of EV-based therapies in oral and maxillofacial reconstruction. Despite the promising mechanistic evidence, most studies investigating these signaling pathways have been conducted *in vitro* or in small animal models, limiting their clinical applicability. Furthermore, multiple signaling pathways often interact in a context-dependent manner, making it difficult to attribute regenerative effects to a single pathway. Differences in EVs source, cargo composition, isolation techniques, dosage, and delivery strategies further contribute to variability in biological responses and limit reproducibility across studies. Consequently, standardized EV production protocols, mechanistic studies in clinically relevant models, and well-designed clinical trials are needed to validate the safety, efficacy, and long-term therapeutic potential of EV-mediated signaling in oral and maxillofacial regeneration.

### 3.2 Bioactive molecules and signaling mediators carried by EVs

The regenerative effects of extracellular vesicles are largely attributed to their cargo of bioactive molecules, including microRNAs (miRNAs), long non-coding RNAs (lncRNAs), messenger RNAs, proteins, lipids, and growth factors (Li M. et al., 2022; Lee and Kim, 2021). These molecules function as signaling mediators that influence recipient cells and regulate tissue repair processes within the oral and maxillofacial region.

Among the most studied EVs cargoes are microRNAs, which act as post-transcriptional regulators of gene expression. Several osteogenesis-associated miRNAs, including miR-21, miR-26a, miR-29, miR-135b, miR-196a, miR-381, and miR-2861, enhance osteoblastic differentiation through activation of Wnt, BMP, and PI3K/Akt pathways (Dai and Dai, 2026; Ráez-Meseguer et al., 2026). In jawbone regeneration, EVs-associated miR-381 has been shown to promote osteogenic gene expression and bone formation. Similarly, miR-21-5p contributes to tissue repair by reducing apoptosis and enhancing angiogenesis (Wang B. et al., 2025).

Long non-coding RNAs such as MALAT1 also participate in regenerative signaling by modulating osteogenic differentiation and bone remodeling (Peng S. et al., 2018). In addition, EVs contain proteins involved in mineralization and matrix formation, including RUNX2, alkaline phosphatase (ALP), osteocalcin (OCN), osteopontin (OPN), bone morphogenetic proteins (BMPs), and collagen-related proteins (Li M. et al., 2022; Biswas et al., 2025). These molecules directly stimulate bone matrix deposition and tissue maturation.

Growth factors packaged within EVs, including VEGF, FGF, TGF-β, and platelet-derived growth factor (PDGF), enhance angiogenesis and cellular recruitment (Matsui and Tabata, 2012; Ko and Naora, 2020). In pulp-dentin regeneration, EVs promote endothelial cell proliferation and vascular network formation through VEGF-related signaling. Collectively, these diverse molecular cargoes form a sophisticated communication system that enables EVs to orchestrate bone, periodontal, pulp, cartilage, nerve, and soft tissue regeneration, making them promising candidates for future cell-free therapies in maxillofacial tissue engineering.

The EVs, which are loaded with genetic materials, share unique biological interactions between the host cell and different microorganisms that play a role in maintaining the harmonious oral microbiota (Yáñez-Mó et al., 2015; Fang et al., 2022). The specific EVs are released from different host cells, such as gingival fibroblasts, osteoblasts, immune cells, epithelial cells, and immune cells express cell signals to those released by common microorganisms like bacteria and fungi, thus contributing to maintaining the oral cavity homeostasis through inter-kingdom interaction (Di Naro et al., 2025). Various types of communications are described as follows.

Host–Microbe Communication: The oral cavity cells, especially the fibroblast immune-presenting cells, express diverse immune-modulating biomolecules such as microRNAs, several cytokines, etc., which influence the microbiota of the oral cavity through modulation of gene expression and alteration of the virulence nature of the bacteria and fungi (Moreno et al., 2023). This two-way interaction of cell signaling through EVs expression provides a better understanding of maintaining the immune regulation of the oral cavity. This host-microbe communication through EVs regulates bacterial aggregation or biofilm formation and even induces immune responses, thus shaping oral health (Wang J. et al., 2024).

Microbe–Host Communication: In contrast to the host exosomes, a subclass of EVs that form an important part of EVs in signal modulation, these nanoparticles released from the microbial organisms present in the oral cavity in diverse diseases also affect immune system activation, thus proving the protective mechanism against pathogenic cell signaling (Macia et al., 2019). Pathogenic microorganisms like *Porphyromonas gingivalis* and *Candida albicans* release EVs loaded with pathogenic proteins, lipids, virulence biomolecules, etc., which target specific receptors, thus leading to a strong immune response from the host cell (Wronowska et al., 2025; Wu et al., 2025). Additionally, the fungal EVs can regulate the microbial biofilm formation of both fungal and bacterial species in the oral cavity, thus explaining the complex interaction in symbiosis or competitive phenomena. This reveals in-depth complex cross-kingdom communication via EVs expression.

Bidirectional Communication in the Oral Cavity: The bidirectional nature of the cellular EVs facilitates cross-kingdom communication by modulating the signaling biomolecules between the host and oral microbiomes, which form unique microbial communities (Correa et al., 2020). Additionally, bacterial outer membrane vesicles (OMVs) containing exosomes regulate *C*. *albicans* biofilm formation, and in turn, fungal exosomes representing EVs influence bacterial colonies (Correa et al., 2020). This cross-kingdom cellular interaction through extracellular vesicle expression provides key understandings in influencing immune responses, microbial behavior, and disease progression.

**FIGURE 3:**
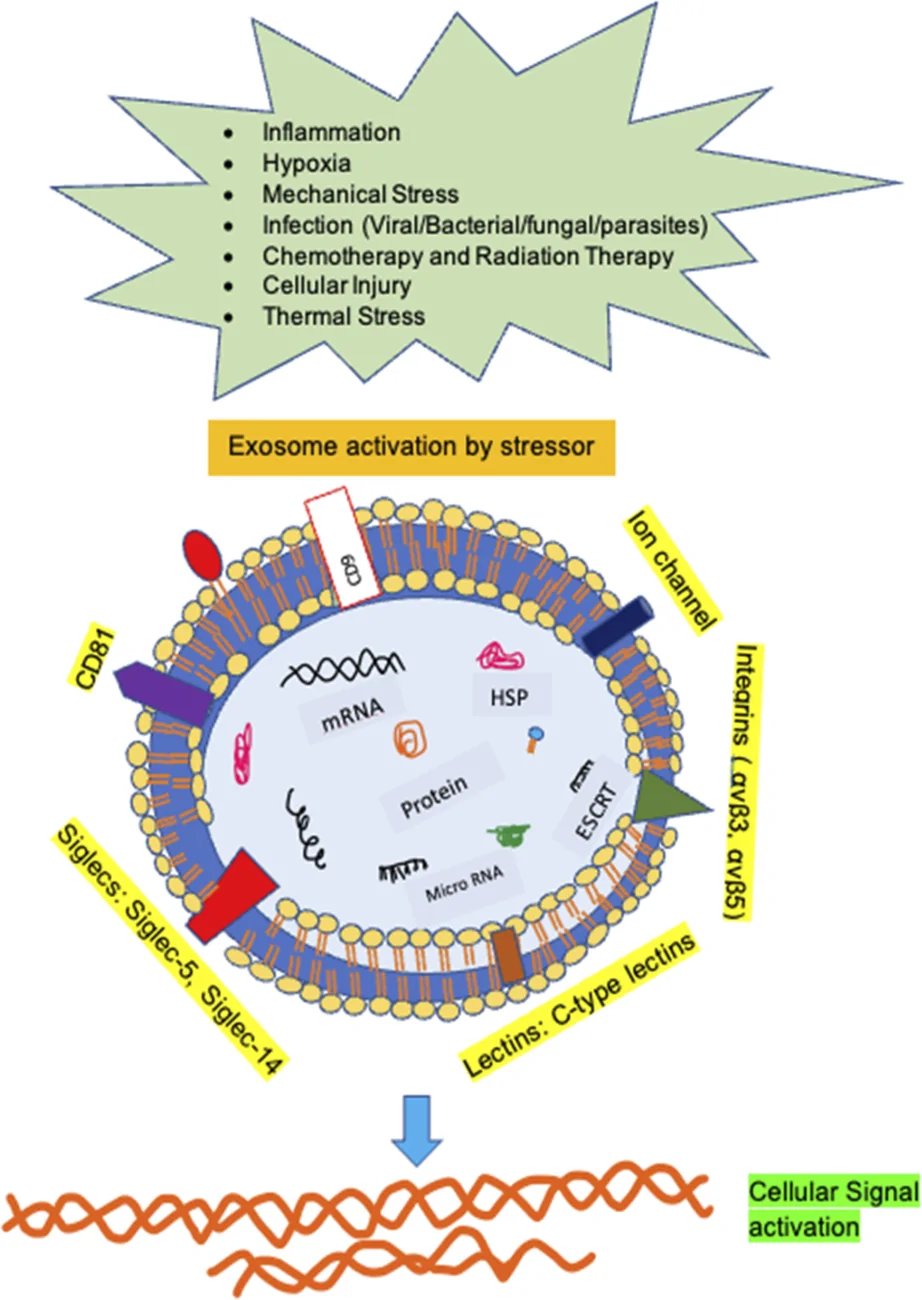
Structure and functions of EVs. Upon binding to target receptors, these nanoparticles transfer bioactive molecules that trigger physiological responses. Figure created on Microsoft PowerPoint 2021 (Microsoft Corp., Redmond, Washington, United States).

EVs are involved in transferring biomolecules that modulate gene expression, inflammation, and tissue regeneration. After the onset of various triggers like stress, inflammation, tissue injury, hypoxia, etc., these nanoparticles are released from the cells and then transported to the target tissue for cellular communication, activated by different receptors. They are supposed to be loaded with biologically active substances like proteins, lipids, and nucleic acids secreted by cells, thus playing a role in various physiological and pathological processes through autocrine and paracrine mechanisms (Chevillet et al., 2014). Their role in cellular communication has attracted significant interest in regenerative medicine in different fields. The genetic material contained in the EVs could alter the cellular function in both positive and negative ways (Panfoli and Bruschi, 2020). One of the shortcomings is that the mutation of the genetic material packed in EVs could degrade the metabolic process of the distant cells/tissues and ultimately either lead to cellular death or proliferation of defective cells in an uncontrolled manner, such as oncogenesis, as shown in Figure 3.

Upon binding to target receptors, these nanoparticles transfer bioactive molecules that trigger physiological responses such as regulation of gene expression, cell proliferation, wound healing, growth and development, cell signaling, cellular apoptosis, angiogenesis, cellular waste management, tissue repair and regeneration, tumorigenesis regulation, and immune modulation (Paskeh et al., 2022). The cargo material packed within the EVs can be genetically modified and transformed to transform the cellular signal to target cells for cellular modification. Such important properties of the nanoparticles can be applied in targeted drug therapy, altering genetic mutation, cellular proliferation, strengthening cellular function, ameliorating the unwanted cellular process, and reducing cellular senescence in regenerative medicine.

In regenerative dentistry, EVs derived from dental pulp stem cells have demonstrated significant therapeutic potential. Their cargo, including VEGF, FGF, BMPs, and regenerative miRNAs, promotes angiogenesis, odontogenic differentiation, and immunomodulation, ultimately supporting dentin–pulp complex regeneration. Consequently, EVs-based therapies have emerged as promising cell-free approaches for tissue engineering and regenerative medicine.

The Minimal Information for Studies of Extracellular Vesicles (MISEV) guidelines, published by the International Society for Extracellular Vesicles (ISEV) provided an updated snapshot of available approaches and their advantages and limitations for production, separation, and characterisation of EVs from multiple sources, including cell culture, body fluids and solid tissues. These recommendations improved methodological rigor, transparency, reproducibility, and comparability across studies. The MISEV 2023 (MISEV 2023) guidelines updated previous recommendations to reflect advances in EV research while reinforcing the need for rigor, transparency, and standardized reporting (Welsh et al., 2024). MISEV2023 recommends that EV isolation methods be selected according to the intended application and that researchers report all pre-analytical variables, isolation procedures, and purification steps in sufficient detail. Because no isolation method yields completely pure EVs, the guidelines encourage assessing both EV recovery and contaminating non-EV components and, where appropriate, combining complementary isolation strategies.

For EV characterization, MISEV2023 emphasizes a multimodal approach that includes evaluation of particle size and concentration, morphology, and molecular composition. The guidelines recommend demonstrating the presence of EV-associated proteins alongside markers of potential contaminants and encourage quantitative reporting to improve data quality and comparability.

Regarding nomenclature, MISEV2023 continues to recommend the generic term EVs unless the vesicle biogenesis has been conclusively demonstrated. When possible, EVs should be further described according to physical characteristics (e.g., size or density), biochemical composition, cellular source, or experimental conditions rather than using terms such as “exosomes” or “microvesicles” without sufficient evidence.

To enhance reproducibility, MISEV2023 promotes detailed reporting of experimental design, sample collection, storage conditions, isolation workflows, characterization methods, functional assays, controls, and data analysis. The guidelines also encourage the use of community resources and transparent reporting standards to facilitate replication and comparison across laboratories.

These recommendations also improve the interpretation of published studies by providing criteria to assess methodological quality, EV purity, and experimental limitations. Consequently, researchers and readers can more confidently distinguish robust biological findings from artifacts caused by contaminants or insufficient characterization, thereby strengthening the translation of EV research into diagnostic and therapeutic applications.

Earlier studies often lacked adequate characterization or purity assessments, making comparisons difficult and occasionally leading to overinterpretation of biological effects. Applying MISEV criteria allows readers to critically assess study quality, distinguish robust findings from methodological artifacts, and improve confidence in reported EV functions and clinical applications.

## 4 Applications of extracellular vesicles in medicine

EVs have demonstrated significant potential in the treatment of a variety of diseases, including wound healing, oxidative stress, graft-versus-host disease, dermatitis, psoriasis, cutaneous cancer, oral and periodontal diseases, as well as cosmetic applications such as wrinkle reduction, scar healing, and hair rejuvenation in cases of alopecia (Thakur et al., 2023; Norouzi et al., 2024). However, it is important to recognize that EVs therapy can have a dual effect (Hazrati et al., 2022). In some instances, the use of achieving the intended therapeutic outcomes may have a negative impact on the targeted tissue, thereby leading to the production of unhealthy tissue or cellular death. This adverse effect may occur during the transmission of signals by diseased parenchymal cells. EVs thus can bring harmful biological signals, thereby disrupting the normal cellular homeostasis, leading to pathological responses in the targeted tissue. These aberrant signals, when transmitted between cells, can contribute to conditions such as oncogenesis, where extracellular vesicles carrying tumorigenic signals may facilitate distant metastasis, thereby exacerbating the spread of cancer to healthy tissues (Li et al., 2019; Mao et al., 2019). Various applications are described as follows.

### 4.1 In tissue regeneration

Various growth factors, notably BMPs, such as BMP-2 and BMP-7, play an integral part in osteogenesis. Dental EVs coated with BMPs bind to specific receptors on dental pulp cells or periodontal ligament fibroblasts, activating the Smad signaling cascade and thus upregulating genes associated with odontoblast development. Similarly, another growth factor, especially VEGF, acts on endothelial cells by activating the PI3K/Akt signaling pathway, hence promoting endothelial cell proliferation and migration. This stimulates the formation of new blood vessels in the dental pulp, enhancing the transport of nutrients and oxygen essential for tissue regeneration. FGFs trigger the MAPK/ERK pathway, a crucial signaling cascade implicated in cell proliferation and differentiation (Zou et al., 2023). In dental tissues, FGF can enhance the regeneration of periodontal ligament cells and stimulate dental pulp stem cells. TGF-β demonstrates dual function in tissue regeneration. It can enhance collagen synthesis in dental tissues and reduce fibrosis, while also modulating odontoblast differentiation.

### 4.2 In tissue inflammation

Several cytokines involved in inflammation, especially IL-1 and TNF-α, are transported by exosomes, one of the important subclasses of EVs that interact with the corresponding receptors on dental pulp cells, activating the NF-κB signaling pathway (Li K. L. et al., 2022; Zeng et al., 2024). This promotes the synthesis of additional cytokines and inflammatory mediators, enhancing the inflammatory response. EVs can reduce or increase the anti-inflammation cytokines depending on the originating cells (Bahri et al., 2024). Oral administration of Ginger-Derived Nanoparticles 2 (GDNPs 2) increases the survival and proliferation of intestinal epithelial cells and reduces the proinflammatory cytokines (TNF-α, IL-6, and IL-1β) and increases the anti-inflammatory cytokines (IL-10 and IL-22) in colitis models. The synthesis of cytokines and inflammatory mediators leads to the recruitment of immune cells, particularly macrophages and neutrophils, to the site of damage or infection; if inflammation continues, it may lead to disorders such as pulpitis or periodontal disease. Likewise, IL-6, transported by extracellular vesicles, promotes the JAK/STAT3 signaling pathway, enhancing the production of acute-phase proteins and immunological responses (Deng et al., 2025). Increased IL-6 concentrations in dental tissues exacerbate chronic inflammation, disturbing the equilibrium of periodontal and pulp tissues. Matrix metalloproteinases (MMPs), including MMP-1 and MMP-9, are enzymes contained in dental exosomes that dissolve collagen fibers and other extracellular matrix (ECM) components in dental tissues (Elgezawi et al., 2022). Thus, EVs carry a signal for matrix remodeling, which is crucial for regular tissue turnover; nonetheless, increased MMP activity can result in tissue degradation in conditions such as periodontal disease or root resorption.

### 4.3 As an extracellular matrix modulator

Collagen is a fundamental structural protein in oral and dental tissues, and EVs and their subclasses containing collagen precursors or remodeling enzymes, such as Collagenase (MMP-1), are integral to collagen turnover, regulating tissue healing and homeostasis. In circumstances of periodontal disease or trauma, significant degeneration of collagen may occur, potentially compromising the structural integrity of the tissue. Fibronectin, a glycoprotein that interacts with integrins, enhances cell mobility and adhesion. Fibronectin embedded in the cellular EVs might influence the migration and adhesion of fibroblasts, hence facilitating periodontal repair and tissue regeneration. Integrins facilitate cell adherence to the extracellular matrix, hence enhancing cell motility and differentiation. Dental EVs containing integrin-binding proteins participate in wound healing, activate signaling pathways, and promote matrix remodeling in dental tissues. Tissue Inhibitors of Metalloproteinases (TIMPs) modulate MMP activity, hence facilitating appropriate matrix decomposition. EVs containing TIMPs also prevent the excessive degradation of the ECM, preserving tissue integrity and facilitating effective ECM remodeling during the repair process.

Although EVs have demonstrated considerable potential for tissue regeneration, immunomodulation, extracellular matrix remodeling, and the treatment of oral and systemic diseases, current evidence remains predominantly derived from *in vitro* studies and preclinical animal models, limiting direct clinical translation. Moreover, the therapeutic effects of EVs are influenced by their cellular origin, donor variability, cargo composition, isolation and purification methods, dosage, delivery strategies, and interactions among multiple signaling pathways, contributing to inconsistent outcomes across studies. While EVs generally promote tissue repair and regeneration, those derived from diseased cells may also transmit pathological signals, potentially exacerbating inflammation, fibrosis, or tumor progression. Consequently, standardized manufacturing and characterization protocols, comprehensive safety assessments, mechanistic studies, and well-designed clinical trials are essential to establish the long-term efficacy, safety, and clinical applicability of EV-based therapies in regenerative medicine and oral and maxillofacial reconstruction.

## 5 Applications of extracellular vesicles in dentistry

Even though the use of extracellular vesicles in other fields of medicine has been extensively studied, their use in dentistry remains low (Xia et al., 2024; Wang et al., 2023). With strong evidence in regenerative medicine, the researchers have expanded the growing interest in tissue regeneration in the pathologically affected dental tissue. Very few scientific contributions have been mentioned in the field of modern dentistry. The transformative role of EVs includes regenerative therapies, improving dental implant integration, aiding diagnostics, reducing inflammation, and enabling targeted drug delivery for various oral diseases. Their diverse applications are advancing both existing and novel treatment strategies in dentistry.

### 5.1 Tissue regeneration

EVs are explored for their potential to regenerate damaged tissues in different diseases like craniofacial defects, oral inflammatory diseases, oral cancer, etc., in dentistry. The healing and cell rejuvenation properties of these nanoparticles can be utilized for tissue regeneration. EVs derived from stem cells are being investigated for their ability to repair dental pulp tissue, periodontal tissues, and bone defects in endodontics. Moreover, MSCs expressing EVs can regulate inflammation in dental tissue by delivering anti-inflammatory proteins, such as IL-10, and by influencing regulatory T cells (Tregs) (Kong et al., 2024). Additionally, a prominent subclass of EVs called exosomes contains microRNAs that modulate gene expression in dental tissue, thereby promoting healing and tissue repair. Odontogenic EVs, by mimicking the natural developmental microenvironment, have the remarkable ability to promote the complete regeneration of the pulp-dentin complex, orchestrating tissue repair and restoration with precision and fidelity (Wang Y. et al., 2025). Thus, EV-based biological therapies can enhance wound healing by stimulating cell migration, proliferation, and tissue angiogenesis (Li B. et al., 2022). They are being studied for applications in periodontitis treatment, pulp regeneration, and bone healing after dental implants.

### 5.2 Disease diagnosis

**FIGURE 4:**
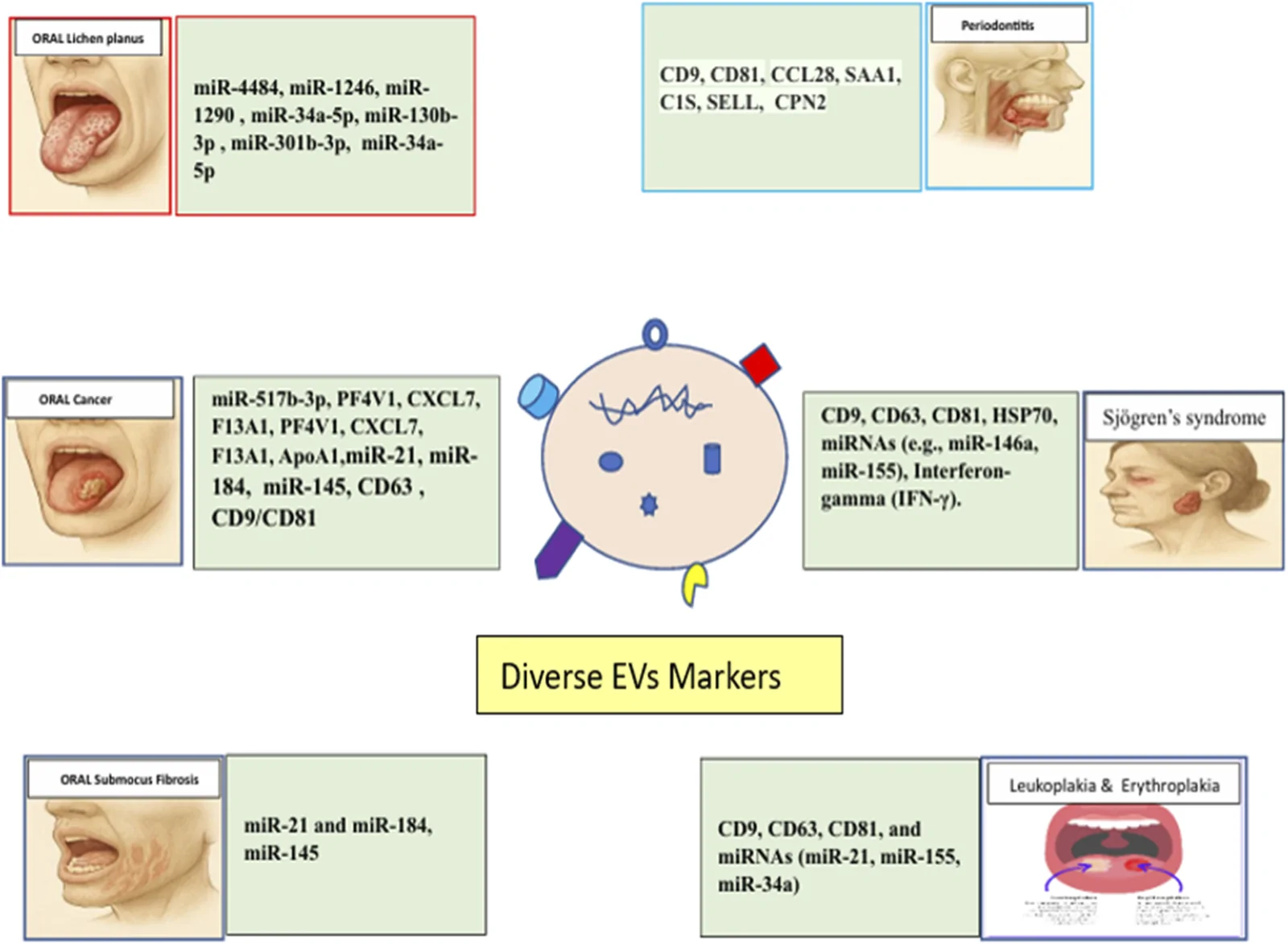
Extracellular vesicles (EVs) markers in diagnostic applications in dentistry. EVs play a crucial role in identifying a range of pathological conditions within the oral cavity: periodontal disease, oral lichen planus, Sjögren’s syndrome, and precancerous lesions such as leukoplakia, erythroplakia, and oral submucous fibrosis. Figure created on Microsoft PowerPoint 2021 (Microsoft Corp., Redmond, Washington, United States).

There is also growing interest in using EVs as diagnostic tools in disease diagnosis. Biomarkers present in extracellular particles could potentially help in detecting periodontal disease, oral cancers, or other oral conditions at an earlier stage with more accuracy (Stanko et al., 2018; Haidar, 2018). EVs derived from the oral cavity express pathological markers released in diverse pathological conditions, which in turn help in the early diagnosis as well as monitoring therapeutic response as diagnostic and prognostic markers for various oral diseases, including periodontitis, primary Sjögren’s syndrome, oral mucosal diseases, hand-foot-mouth disease, and oral malignancies, as shown in Figure 4. These nanoparticles are loaded with genetic materials that signal disease modification, such as periodontitis, Oral lichen planus, Sjogren syndrome (Cui et al., 2025), and oral cancer (Xing et al., 2020; Huang Y. et al., 2020; Byun et al., 2015). The EVs released from the diseased cells are often loaded with the mutated genetic materials (RNA/DNA/proteins), which can be diagnosed by different methods in the laboratory. The biomarkers contained in these vesicles, derived from the saliva, such as CD9 and CD81, can help to diagnose cases of periodontitis through ELISA methods (Tobón-Arroyave et al., 2019). In addition to this, other markers such as CCL28, SAA1, C1S, SELL, and CPN2 are critically implicated in the inflammatory and immune processes associated with periodontitis; diagnosis through a combination of clinical examination, molecular analyses of proteins, and biomarker profiling from saliva or GCF (Huang X. et al., 2020). These EVs markers provide valuable insights into the pathophysiology of periodontal disease, offering potential applications in diagnosis, monitoring, and understanding disease progression. Their presence signifies ongoing immune activation and tissue damage, both of which are hallmark features of periodontitis.

Previous studies have shown that miR-4484, miR-1246, and miR-1290 were found to be upregulated in salivary extracellular vesicles of OLP patients (Huang X. et al., 2020). Similarly, in plasma-derived EVs and their exosomes from OLP patients, miR-34a-5p and miR-130b-3p were up, and miR-301b-3p was down, and miR-34a-5p correlated positively with the disease severity (Peng Q. et al., 2018). Another study of plasma circulating EVs microRNAs in patients with OLP, Oral Submucous Fibrosis (OSMF), and combined lesions revealed that there was upregulation of miR-21 and miR-184, while miR-145 was downregulated in the case group (OLP/OSMF) vs. healthy controls (Yasothkumar et al., 2024). Such genetic markers of cell-derived EVs can be studied for diagnostic purposes in dentistry. One of the important discoveries that can benefit the near future is that the earliest diagnostic markers are often loaded in different types of EVs, which are continuously released before the disease’s progression approaches the advanced stage. So, these nanoparticles confer earlier diagnostic benefits as well as disease monitoring, apart from tissue regeneration potential.

Sjögren’s syndrome (SS) is a chronic autoimmune disease that primarily affects the salivary and lacrimal glands, leading to dryness of the mouth and eyes can be diagnosed through salivary EVs markers like CD9, CD63, and CD81, HSP70 (Heat Shock Protein 70), miRNAs (e.g., miR-146a, miR-155), and Interferon-gamma (IFN-γ), which are expressed in diseases condition. (Yasothkumar et al., 2024; Gao et al., 2018; Chang et al., 2019). Similarly, another precancerous lesion, like leucoplakia, that has a chance of disease progression to oral squamous cell carcinoma might be detected through EV markers. The study of salivary EVs in leukoplakia provides extensive insights into early detection, malignant transformation, and disease progression. CD markers on these nanoparticles, like CD9, CD63, and CD81, miRNAs (e.g., miR-21, miR-34a, miR-145), VEGF, Epidermal Growth Factor Receptor (EGFR), etc., can be utilized for the diagnosis of a variety of diseases (Jiang et al., 2017; De Waele et al., 2020; Joshi et al., 2018; Zhang and Wang, 2019; Malla et al., 2018).

### 5.3 In early detection of oral cancer

Oral cavity cancer continues to present significant public health challenges, with a substantial increase in affected individuals expected in the coming decades (Alves-Costa et al., 2025). These trends highlight the need for targeted prevention, improved early detection, and policies addressing modifiable risk factors to reduce the projected burden in high-risk populations. Oral squamous cell carcinoma (OSCC), one of the most common oral cancers, is often diagnosed at a late stage and has a very poor prognosis. Salivary EVs have demonstrated promising biomarkers for the early detection of OSCC, offering potential for improved outcomes (He et al., 2020; Guo et al., 2021; Nakamichi et al., 2021). EVs are ubiquitous, being secreted by nearly all cell types in the body, including both healthy and diseased tissues. These vesicles encapsulate bioactive molecules that mirror the condition of their source, rendering them valuable biomarkers for tracking disease progression, evaluating therapeutic responses, and detecting metastasis (LeBleu and Kalluri, 2020). The EVs from oral cancer cells play a role in tumor progression, immune evasion, and metastasis. They can transfer oncogenic signals to neighboring cells, promoting cancer cell proliferation, invasion, and drug resistance. The infected cancer cells release different substances like CD81, CD9, CD63, miR-302b-3p, miR-517b-3p, Platelet Factor 4 Variant 1 (PF4V1), C-X-C Motif Chemokine Ligand 7 (CXCL7), Coagulation Factor XIII A Chain (F13A1), Apolipoprotein A1 (ApoA1), loaded in EVs of defective genes that can be one of the important clues to the tumorigenesis (Xing et al., 2020). Similarly, the EVs extracted from the oral squamous cell cancer from the patient, when compared with the samples from healthy controls, demonstrated an increased concentration and larger diameter of CD63 and lower CD9/CD81 expression levels, thus helping in the early diagnosis of oral cancer before it becomes clinically apparent (Zlotogorski-Hurvitz et al., 2016). These genetic materials can be studied, and different types of oral cancer can be checked and resected at the earliest stage. The diagnostic marker for the different cancers studied from these nanoparticles present in salivary fluid, plasma, and blood can be one of the new eras of studies in disease identification and therapeutic areas in the field of oral cancer in modern dentistry.

### 5.4 Drug delivery platform

EVs may also serve as a therapeutic tool by delivering drugs (Lee et al., 2022) or RNA molecules directly to cancer cells, potentially improving the precision and efficacy of cancer treatments (Yong et al., 2019). In dentistry. EVs-coated drug delivery in dentistry offers a promising approach for targeted and controlled treatment of oral diseases. Drugs can be encapsulated inside EVs or attached to their surface, allowing for precise delivery to specific oral tissues, such as the periodontal ligament or dental pulp (Yong et al., 2019). EVs can naturally cross biological barriers, enabling efficient delivery to hard-to-reach areas like inflamed gums or deep dental tissues. By modifying the surface of EVs and their exosomes with ligands or antibodies, they can be directed to target specific disease markers, such as in periodontal disease or oral cancers.

The approach of chemotherapy introduction through genetically engineered modified EVs loaded with chemotherapy has been proven to be an important milestone in the drug administration to patients with diverse cancers (Li et al., 2020; Zhang et al., 2021). Such therapies provide superior targeted efficacy in drug administration (Yong et al., 2019). Additionally, EVs provide sustained and controlled release of therapeutic agents, reducing the need for frequent administration and enhancing long-term therapeutic efficacy. It is also noteworthy to consider that the successful trial has been made with the introduction of exosome-based chemotherapy for many cases of chemotherapy-resistant cancer patients (Yang et al., 2017; Yang et al., 2018). With successful experiments on many cancer patients, the EVs can be genetically modified and packed with chemotherapy agents for the management of different types of oral cancer, including chemotherapy-resistant cases, in the field of future dentistry.

Despite the expanding applications of EVs in dentistry, several challenges hinder their translation into routine clinical practice. Most current evidence is derived from laboratory and animal studies, with relatively few well-designed human clinical trials demonstrating long-term efficacy and safety. Furthermore, the diagnostic and therapeutic performance of EVs is influenced by variability in their cellular origin, isolation and purification techniques, cargo composition, storage conditions, and delivery strategies, resulting in inconsistent findings across studies. While EVs offer promising opportunities for tissue regeneration, disease diagnosis, early cancer detection, and targeted drug delivery, concerns remain regarding scalability, manufacturing under Good Manufacturing Practice standards, biodistribution, immunogenicity, and the potential transmission of pathogenic or oncogenic signals. In addition, many proposed EV biomarkers require validation in large, multicenter cohorts before they can be reliably implemented in clinical diagnostics. Future research should prioritize standardized protocols, mechanistic investigations, and randomized controlled clinical trials to establish the safety, reproducibility, cost-effectiveness, and clinical utility of EV-based technologies in modern dentistry.

## 6 Introduction of extracellular vesicles into biological systems

Another step in the therapeutic application of EVs in Dentistry is their delivery to the targeted tissue. Different methods are introduced for the introduction of EVs particles in different areas of regenerative dentistry to ensure the précised and desired clinical outcome. The laboratory-prepared cell-derived EVs can be introduced to the regenerative tissue through the following routes.

### 6.1 Systemic injection of EVs in drug delivery

This is one of the easily accessible methods of EVs introduction to targeted dental tissue in the field of regenerative dentistry. Previous studies have demonstrated the EV-mediated therapeutic effect after systemic injection of targeted extracellular vesicles in animal models (Alvarez-Erviti et al., 2011). The clinical procedure is like other drug introductions through the intravenous route in regenerative medicine. While direct studies on intravenous EVs particle delivery specifically for dental tissue regeneration are limited, existing research supports the potential of systemic extracellular nanoparticle administration in enhancing dental tissue repair. In a rat model study on bisphosphonate-induced osteonecrosis (Wang et al., 2019), the introduction of healthy EVs through the intravenous route modulated the gene expression, thereby contributing to the newborn formation in the maxilla. These discoveries paved the way for the clinical application of EVs-based regenerative procedures in modern dentistry. Although it’s a feasible technique for EVs’ introduction, the reduction of the concentration of nanoparticles is the major drawback. Apart from this, it might be difficult to estimate the precise concentration required to reach the target tissue with blood and achieve the targeted therapeutic goal. Another issue might arise from the necessity of autologous EVs particles to prevent an immune reaction.

### 6.2 Targeted EVs delivery through local injection

Another commonly employed method of drug delivery can also be used for the introduction of EVs to the targeted tissue. With the availability of desired EVs and their exosomes, these nanoparticles can be introduced to the different deformed dental tissues, such as the pulp cavity, PDL, gingiva, maxilla, mandible, etc. The introduction of the skin rejuvenation procedure might be a better option for the healing of chronic inflammatory lesions like lichen planus, non-healing oral ulcers, including aphthous ulcers, since previous studies have demonstrated satisfying results in chronic diabetic ulcers (Li et al., 2018). Still, there are no published reports of EVs in lichen planus. Chronic non-healing ulcers can benefit from these nanoparticles’ application directly into the targeted tissue in the outpatient department. Similarly, EVs can be utilized for the early and better regeneration of the flap in oral-facial reconstruction (Xie et al., 2019). This method might be easier for dental practitioners. One of the main shortcomings in such EVs delivery might be the frequent introduction at regular intervals, as well as some amount of wastage during injection into the dental tissue. Some case reports have also revealed tissue necrosis after tissue injection for EVs for therapeutic intervention, which is another drawback, but the incidence is very low (Tawanwon et al., 2024; AlBargawi, 2025).

Shimizu et al. (Shimizu et al., 2022), studied the effect of local administration of haplotype homo (HHH)-DPC exosomes in a mouse model of periodontitis. They found that treatment with HHH-dental pulp cells (DPC) exosomes did not alter the differentiation of osteoblastic cells. µCT images showed that the exosomes suppressed alveolar bone resorption in the mouse model of periodontitis. Although no change was apparent in the dominance of TRAP-positive osteoclast-like cells in decalcified tissue sections upon exosome treatment, HHH-DPC exosomes significantly suppressed osteoclast formation *in vitro*. They concluded that the HHH-DPC exosomes stimulated the migration of human DPCs and mouse osteoblastic cells and effectively attenuated bone loss due to periodontitis.

### 6.3 Innovative carrier systems for EVs-Mediated drug delivery

Another method that might be employed for the sustained release of extracellular vesicles for tissue regeneration in dentistry is the use of carrier-based delivery systems, such as ceramics and hydrogels (Hwang and Lee, 2024). Unlike injection delivery methods of EVs application, these methods offer significant advantages in controlled release and prolonged retention of exosomes, thus making them one of the ideal methods for soft tissue regeneration in deformed dental pulp and better augmentation in therapeutics (Hwang and Lee, 2024; Ju et al., 2023). Apart from this, other synthetic polymers such as PLGA provide a more predictable and sustained release of EVs, further promoting tissue regeneration (Yang et al., 2022). On the other hand, ceramics, particularly hydroxyapatite and beta-tricalcium phosphate, are effective in hard tissue regeneration, thus contributing to osteogenesis (Brochu et al., 2024; Skallevold et al., 2019). However, challenges like burst release and limited long-term exosome delivery remain visible. The combination of these vesicles with hybrid scaffolds (such as natural collagen-based scaffolds and synthetic/hybrid materials) may offer superior regenerative potential, though further research is required for validation. These recent advancements have the potential to significantly impact dental regenerative therapies in modern regenerative dentistry.

## 7 Current clinical status and future directions of extracellular vesicles in dentistry

Studies demonstrating the cancer marker nature of dental EVs have continued to expand in a progressive manner even nowadays (Qoronfleh and Al-Dewik, 2025). Similarly, there are also human studies where EVs derived from saliva and gingival crevicular fluid have demonstrated the potential markers of disease burden in the case of periodontitis (Cai et al., 2023; Wu et al., 2023). EVs-based therapies offer the potential for highly personalized dental treatments, especially in regenerative procedures and cancer therapies (Xia et al., 2024). By customizing EVs formulations based on a patient’s specific oral health needs, such as targeted drug delivery to infected or damaged tissues, dental professionals could enhance treatment outcomes and promote more precise, individualized care for conditions like periodontal disease, pulpitis, or oral tumors.

Since the EVs-based tissue regeneration therapies’ holistic combined approach of the EVs with the various dental implants can be another newer treatment modality in dental science in the near future (Yu C. et al., 2024). The implants embedded in the maxillary and mandibular regions, along with other facial bones, can lead to tissue damage to neighboring structures like ligaments, bone, and other soft tissues, which can be one of the important causes of implant removal. These particles are being studied for their ability to enhance bone regeneration around dental implants. EVs derived from MSCs can enhance osteoblast differentiation and improve new bone formation and tissue healing, leading to better implant integration and stability (C et al., 2025).

At present, biological therapies are more commonly offered by many private clinics in the form of injectable and topical. But to date, there is no FDA approval for use in any form, even though pre-clinical studies have shown therapeutic effects. This lack of clarity is primarily due to the absence of established regulatory standards (Yang et al., 2020; Wang C. K. et al., 2024). Despite this, some clinics offer injectable biological EVs therapies, often marketed as “regenerative” or “stem cell-like” treatments (Wang C. K. et al., 2024). The therapeutic significance of EVs therapies in dentistry is still evolving, with many potential uses at various phases of clinical advancement. Various research studies and clinical trials about the use of EVs *in vivo* in most animals have yielded quite satisfactory results. A Phase I/II early clinical trial is now investigating the potential use of EVs and their exosomes manufactured from adipose stem cells as an adjuvant treatment for periodontitis in conjunction with scaling and root planing (Xing et al., 2020). This represents one of the primary attempts to analyze EVs in a human dental context. Regarding periodontal regeneration, research is predominantly preclinical, with substantial evidence from various animal and laboratory studies (Xing et al., 2020). Dental pulp regeneration is predominantly in the *in vitro* and animal clinical trial phases, with research indicating that EVs produced from dental stem cells may encourage dentin-like tissue development and enhance regenerative results (Zhang et al., 2016). Investigations into dentin-pulp complex regeneration remain preclinical, with encouraging results from animal studies indicating the potential of these vesicles to facilitate the regeneration of the pulp-dentin complex (Miao et al., 2025). Finally, experiments regarding applications in orthodontics or bone regeneration remain in the experimental phase in animal models, but no clinical trials have been conducted to assess their complete efficacy in dental practices (Zhang et al., 2016).

EVs markers for diagnosing oral cancer and dental diseases have already moved into human studies**,** although they are still in the early clinical research phase (Zhang et al., 2023). Such markers have been successfully established in human studies to be reliable markers for periodontal disease and oral cancer (OSCC), with correlations to disease severity and significant evidence for noninvasive salivary samples (Hammouda et al., 2023). Hence, EVs and their exosome therapy are still in their early stages, and the industry must tackle ethical challenges related to safety, effectiveness, and regulation to unlock the full potential of exosome-based treatments. Clinical adoption on a large scale is still ongoing.

Finally, continued advancements in technology and experimentation are expected to provide crucial insights into the heterogeneity and biological functions of cell-derived vesicles, while also improving our capacity to leverage their therapeutic and diagnostic potential. Although there are diverse possible applications of EVs in the field of dentistry, the accessibility of these nanoparticles to dentists in every corner of the world is difficult. The standard method of preparation, purity, and quality assessment, and expertise in the laboratory are some of the topmost hindrances to the usage of EVs. While the use of these vesicles in clinical dentistry holds significant promise in the upcoming days, challenges remain, particularly in the standardization of isolation methods, dosages, and formulations. Establishing consistent protocols for EVs extraction from oral tissues, along with optimal drug loading and delivery systems, is crucial for their effective use in dental treatments. Additionally, the safety and long-term effects of EVs-based therapies in oral health require further investigation to ensure their safe implementation in clinical practice.

Despite these encouraging advances, several translational challenges continue to impede the clinical adoption of EV-based therapies in dentistry. The lack of standardized protocols for EV isolation, purification, and characterization remains a major source of variability, as different techniques, including ultracentrifugation, size-exclusion chromatography, polymer-based precipitation, and microfluidic platforms, produce EV populations with differing purity, yield, and biological activity. Furthermore, large-scale manufacturing of clinical-grade EVs under Good Manufacturing Practice conditions remains technically complex and economically demanding because of challenges in maintaining batch-to-batch consistency, sterility, potency, and reproducibility. Comprehensive quality control measures, including standardized assessment of EV identity, purity, particle size distribution, cargo composition, biological activity, and contaminant profiles, are also lacking. Additional concerns include limited knowledge regarding storage stability, optimal cryopreservation methods, shelf life, and preservation of biological function during long-term storage and transportation. From a therapeutic perspective, the biodistribution, pharmacokinetics, tissue targeting, clearance mechanisms, optimal dosage, treatment frequency, and route of administration remain incompletely understood, making dose standardization difficult. Moreover, regulatory approval pathways for EV-based therapeutics are still evolving, with uncertainties regarding product classification, manufacturing requirements, quality assurance, and clinical evaluation. Addressing these challenges will require harmonized international guidelines, robust GMP-compliant manufacturing processes, validated quality control standards, scalable production technologies, and well-designed multicenter clinical trials to ensure the safety, reproducibility, and clinical effectiveness of EV-based therapies in dentistry.

## 8 Limitations of extracellular vesicles applications

Despite having widespread potential applications of cell-released EVs, the use of exosomes is limited. The lack of well-trained manpower, sophisticated machines for EVs production, exosome cauterization, ethical issues, economic burden, etc., has confined the use to only limited settings. Various shortcomings and hindrances to the application of these nanoparticles in modern dentistry, as well as future perspectives, might be challenging. One of the important steps in EVs production is the isolation and purification. Once the stem cells derived from the umbilical cord, DP cells, etc., are chosen, then come the isolating and purifying of EVs and their exosomes procedures, like ultracentrifugation and size-exclusion chromatography (Lu, 2015). Similarly, these procedures also show differences in purity, yield, and miRNA profiles, which can affect the quality of the isolated EVs (De Sousa et al., 2023; Patel et al., 2019). Additionally, these isolation methods are often expensive and typically yield limited quantities of extracellular vesicles, which presents a significant challenge for their widespread clinical application in regenerative dentistry and other therapeutic fields. In addition, the composition of EVs can be influenced by several factors, including the type of cell from which it is extracted, donor age, and the microenvironment in which the parent cells reside (temperature, pH, and rate of metabolism) (Xu et al., 2016; Li et al., 2021). Such variables can significantly influence the therapeutic effect of EVs once applied in targeted tissues. For example, EVs particles derived from MSC (MSC-exosomes) exhibit age-dependent variations (Fafián-Labora et al., 2017; Alibhai et al., 2020), which can affect their therapeutic efficacy in applications like osteogenesis and bone repair (Burrello et al., 2024). These age-related differences highlight the need for a better understanding of how these cell-derived particles as a whole influence their regenerative capacity during tissue regeneration and repair (Xia et al., 2020).

Currently, there is a gap in knowledge and understanding about the standardization in the concentration and dosage of EVs used in various research studies, even though different studies have shown that higher doses generally tend to improve tissue regeneration outcomes (Kalluri and LeBleu, 2020; Lin et al., 2020). This inconsistency in EVs dosage makes it difficult to compare results in different cases and establish reliable therapeutic protocols for the EVs application. It is therefore necessary to set up good manufacturing practices and standardized guidelines for the stem cell extraction, isolation, purification, and application in the targeted tissues (Zhao et al., 2020; Chen et al., 2020).

Another challenge during the use of EVs is the method of sustained release of these microvesicles in the targeted tissue over extended periods to achieve a long-term therapeutic response. To address this issue, the development of EVs-loaded scaffolds with controlled release mechanisms, that is, efficient *in vivo* degradation and high loading capacity, is essential (Thakur and Rai, 2024). Ongoing research is focused on creating advanced delivery systems, such as injectable microspheres (Riau et al., 2019), designed to address tissue defects and conditions like periodontitis, enhancing the effectiveness of EVs-based therapies (Gao et al., 2022). Different methods, like hydrogel, which is commonly used, are often loaded with EVs for sustained and controlled release of these nanoparticles (Chen et al., 2025).

Finally, despite encouraging preclinical findings, the translation of EV-based therapies into routine dental practice remains challenging. One of the major barriers is the lack of standardized protocols for EV isolation, purification, characterization, storage, and quality control, which contributes to considerable variability between studies and limits reproducibility. The optimal source of EVs, therapeutic dose, route of administration, treatment frequency, and long-term biodistribution remain uncertain. Regulatory frameworks for EV-based therapeutics are also still evolving, creating challenges for product classification, quality assurance, and clinical approval. In addition, comprehensive safety evaluations, including assessments of immunogenicity, off-target effects, tumorigenic potential, and long-term biological consequences, are required before widespread clinical adoption. Addressing these translational barriers through multidisciplinary collaboration, standardized reporting guidelines, and well-designed multicenter randomized clinical trials will be essential for realizing the full therapeutic potential of EVs in dentistry.

## 9 Conclusion

EVs have emerged as versatile mediators of intercellular communication with significant implications for regenerative medicine and dentistry. Their ability to transport bioactive molecules—including proteins, lipids, and nucleic acids—enables them to regulate key biological processes such as inflammation, angiogenesis, immune modulation, and tissue repair. In maxillofacial applications, EVs derived from mesenchymal and dental stem cells have shown strong potential in promoting periodontal regeneration, dentin–pulp complex repair, alveolar bone formation, and craniofacial tissue healing. These cell-free approaches offer advantages over traditional stem-cell therapies by reducing immunogenic risks while maintaining potent regenerative capacity. Beyond dentistry, EVs are being explored in oncology, cardiovascular repair, neurology, and targeted drug delivery, reflecting their broad therapeutic relevance. Despite these promising developments, several challenges must be addressed before EVs-based therapies can be translated into routine clinical practice. Standardized protocols for EVs isolation, characterization, storage, and quality control remain essential, along with a deeper understanding of their biodistribution, mechanisms of action, and long-term safety. Advances in scalable manufacturing and regulatory frameworks will further support clinical adoption. As research continues to expand, EVs-based technologies are poised to play a transformative role in future regenerative and precision healthcare strategies.

## Glossary

ApoA1: Apolipoprotein A1
CCL28: C-C Motif Chemokine Ligand 28
C1S: Complement Component 1, S Subcomponent
CD63: Cluster of Differentiation 63
CD81: Cluster of Differentiation 81
CD9: Cluster of Differentiation 9
CXCL7: C-X-C Motif Chemokine Ligand 7
CPN2: Carboxypeptidase N, Polypeptide 2
DFCs: Dental Follicle Cells
DPSCs: Dental Pulp Stem Cells
EGFR: Epidermal Growth Factor Receptor
ELISA: Enzyme-Linked Immunosorbent Assay
ERK: Extracellular Signal-Regulated Kinase
EVs: Extracellular Vesicles
FDA: Food and Drug Administration
F13A1: Coagulation Factor XIII A Chain
GCF: Gingival Crevicular Fluid
GDNPs 2: Ginger-Derived Nanoparticles 2
GMP: Good Manufacturing Practice
hUCMSCs: Human Umbilical Cord Mesenchymal Stem Cells
HSP70: Heat Shock Protein 70
IFN-γ: Interferon-gamma
JAK: Janus Kinase
LPS: Lipopolysaccharide
MAPK: Mitogen-Activated Protein Kinase
MMP: Matrix Metalloproteinase
MVBs: Multi-Vesicular Bodies
MSC: Mesenchymal Stem Cells
miR-302b-3p: MicroRNA-302b-3p
miR-517b-3p: MicroRNA-517b-3p
OLP: Oral Lichen Planus
OSMF: Oral Submucous Fibrosis
PDLSCs: Periodontal Ligament Stem Cells
PI3K/Akt: Phosphoinositide 3-Kinase/Protein Kinase B**.**
PF4V1: Platelet Factor 4 Variant 1
Tregs: Regulatory T cells
SELL: Selectin L (also known as L-selectin)
SS: Sjögren’s Syndrome
STAT3: Signal Transducer and Activator of Transcription 3
TIMPs: Tissue Inhibitors of Metalloproteinases
TLR4: Toll-Like Receptor 4
VEGF: Vascular Endothelial Growth Factor

## References

[B1] Ahmad P. Estrin N. Farshidfar N. Zhang Y. Miron R. J. (2025). Mechanistic insights into periodontal ligament stem cell-derived exosomes in tissue regeneration. Clin. Oral Investig. 29 (7), 357. 10.1007/s00784-025-06422-1 PMC12198077 40562987

[B2] AlBargawi S. (2025). Necrosis following dermal injection of lyophilized exosomes: a case report. J. Cosmet. Dermatol 24 (8), e70387. 10.1111/jocd.70387 40820962 PMC12359287

[B3] Alibhai F. J. Lim F. Yeganeh A. DiStefano P. V. Binesh-Marvasti T. Belfiore A. (2020). Cellular senescence contributes to age-dependent changes in circulating extracellular vesicle cargo and function. Aging Cell 19 (3), e13103. 10.1111/acel.13103 31960578 PMC7059145

[B4] Alvarez-Erviti L. Seow Y. Yin H. Betts C. Lakhal S. Wood M. J. A. (2011). Delivery of siRNA to the mouse brain by systemic injection of targeted exosomes. Nat. Biotechnol. 29 (4), 341–345. 10.1038/nbt.1807 21423189

[B5] Alves-Costa S. Romandini M. Nascimento G. G. (2025). Lip and oral cancer, caries and other oral conditions: estimates from the 2021 global burden of disease study and projections up to 2050. J. Periodontal Res. 60 (6), 544–558. 10.1111/jre.13421 40530949 PMC12312818

[B6] Amin S. Massoumi H. Tewari D. Roy A. Chaudhuri M. Jazayerli C. (2024). Cell type-specific extracellular vesicles and their impact on health and disease. Int. J. Mol. Sci. 25 (5), 2730. 10.3390/ijms25052730 38473976 PMC10931654

[B7] Bahri F. Mansoori M. Vafaei S. Fooladi S. Mir Y. Mehrabani M. (2024). A comprehensive review on ginger-derived exosome-like nanoparticles as feasible therapeutic nano-agents against diseases. Mater Adv. 5 (5), 1846–1867. 10.1039/d3ma00856h

[B8] Berumen Sánchez G. Bunn K. E. Pua H. H. Rafat M. (2021). Extracellular vesicles: mediators of intercellular communication in tissue injury and disease. Cell Commun. Signal 19 (1), 104. 10.1186/s12964-021-00787-y 34656117 PMC8520651

[B9] Bi R. Lyu P. Song Y. Li P. Song D. Cui C. (2021). Function of dental follicle progenitor/stem cells and their potential in regenerative medicine: from mechanisms to applications. Biomolecules 11 (7), 997. 10.3390/biom11070997 34356621 PMC8301812

[B10] Biswas S. Gangadaran P. Dhara C. Ghosh S. Phadikar S. D. Chakraborty A. (2025). Extracellular vesicles in osteogenesis: a comprehensive review of mechanisms and therapeutic potential for bone regeneration. Curr. Issues Mol. Biol. 47 (8), 675. 10.3390/cimb47080675 40864828 PMC12384157

[B11] Brochu B. M. Sturm S. R. Kawase De Queiroz Goncalves J. A. Mirsky N. A. Sandino A. I. Panthaki K. Z. (2024). Advances in bioceramics for bone regeneration: a narrative review. Biomimetics 9 (11), 690. 10.3390/biomimetics9110690 39590262 PMC11592113

[B12] Burrello J. Goi J. Burrello A. Vacchi E. Rendon-Angel A. Lazzarini E. (2024). Age- and sex-related variations in extracellular vesicle profiling for the assessment of cardiovascular risk: the EVaging index. NPJ Aging 10 (1), 63. 10.1038/s41514-024-00189-7 39702460 PMC11659617

[B13] Byun J. S. Hong S. H. Choi J. K. Jung J. K. Lee H. J. (2015). Diagnostic profiling of salivary exosomal microRNAs in oral lichen planus patients. Oral Dis. 21 (8), 987–993. 10.1111/odi.12374 26389700

[B14] Chen M. Huang B. Su X. (2025). Mesenchymal stem cell-derived extracellular vesicles in periodontal bone repair. J. Mol. Med. Berl. 103 (2), 137–156. 10.1007/s00109-025-02513-4 39821702

[B15] Cai R. Wang L. Zhang W. Liu B. Wu Y. Pang J. (2023). The role of extracellular vesicles in periodontitis: pathogenesis, diagnosis, and therapy. Front. Immunol. 14, 1151322. 10.3389/fimmu.2023.1151322 37114060 PMC10126335

[B16] Carvalho Ferraz L. Pereira P. Ferreira J. V. (2025). Molecular mechanisms of extracellular vesicle biogenesis and their impact on the design of custom EVs. Adv. Healthc. Mater 14 (23), e2501349. 10.1002/adhm.202501349 40624965 PMC12417789

[B17] Chang H. X. Tan R. Hartman G. L. Wen Z. Sang H. Domier L. L. (2019). Characterization of soybean STAY-GREEN genes in susceptibility to foliar chlorosis of sudden death syndrome. Plant Physiol. 180 (2), 711–717. 10.1104/pp.19.00046 30952683 PMC6548243

[B18] Chansaenroj J. Kornsuthisopon C. Chansaenroj A. Samaranayake L. P. Fan Y. Osathanon T. (2025). Potential of dental pulp stem cell exosomes: unveiling Mirna-driven regenerative mechanisms. Int. Dent. J. 75 (2), 415–425. 10.1016/j.identj.2024.08.019 39368923 PMC11976581

[B19] Chen Y. S. Lin E. Y. Chiou T. W. Harn H. J. (2020). Exosomes in clinical trial and their production in compliance with good manufacturing practice. Tzu Chi Med. J. 32 (2), 113–120. 10.4103/tcmj.tcmj_182_19 32269942 PMC7137364

[B20] Chen Y. Guo B. Zhang Y. Bao X. Li D. Lin J. (2025). Injectable hypoxia-preconditioned human exfoliated deciduous teeth stem cells encapsulated within GelMA-AMP microspheres for bone regeneration in periodontitis. Colloids Surf. B Biointerfaces 247, 114452. 10.1016/j.colsurfb.2024.114452 39689590

[B21] Chevillet J. R. Kang Q. Ruf I. K. Briggs H. A. Vojtech L. N. Hughes S. M. (2014). Quantitative and stoichiometric analysis of the microRNA content of exosomes. Proc. Nattl Acad. Sci. U. S. A. 111 (41), 14888–14893. 10.1073/pnas.1408301111 25267620 PMC4205618

[B22] Correa R. Caballero Z. De León L. F. Spadafora C. (2020). Extracellular vesicles could carry an evolutionary footprint in interkingdom communication. Front. Cell Infect. Microbiol. 10, 76. 10.3389/fcimb.2020.00076 32195195 PMC7063102

[B23] Cui L. Zheng J. Lu Y. Lin P. Lin Y. Zheng Y. (2024). New frontiers in salivary extracellular vesicles: transforming diagnostics, monitoring, and therapeutics in oral and systemic diseases. J. Nanobiotechnology 22 (1), 171. 10.1186/s12951-024-02443-2 38610017 PMC11015696

[B24] Cui X. Liu L. Duan C. Mao S. Wang G. Li H. (2025). A review of the roles of exosomes in salivary gland diseases with an emphasis on primary Sjögren’s syndrome. J. Dent. Sci. 20 (1), 1–14. 10.1016/j.jds.2024.10.001 39873057 PMC11762945

[B25] Dai Q. Dai Q. (2026). Stem cell-derived exosomes in tissue regeneration of oral and maxillofacial region: a systematic review. Med. Baltim. 105 (1), e46948. 10.1097/MD.0000000000046948 41496030 PMC12778111

[B26] De Sousa K. P. Rossi I. Abdullahi M. Ramirez M. I. Stratton D. Inal J. M. (2023). Isolation and characterization of extracellular vesicles and future directions in diagnosis and therapy. Wiley Interdiscip. Rev. Nanomed Nanobiotechnol 15 (1), e1835. 10.1002/wnan.1835 35898167 PMC10078256

[B27] de Souza W. Gemini-Piperni S. Ruivo C. Bastos N. Almeida S. Lopes D. (2024). Osteoblasts-derived exosomes as potential novel communicators in particle-induced periprosthetic osteolysis. Mater. Today Bio 28, 101189. 10.1016/j.mtbio.2024.101189 PMC11364904 39221219

[B28] De Waele J. J. Boelens J. Leroux-Roels I. (2020). Multidrug-resistant bacteria in ICU: fact or myth. Curr. Opin. Anaesthesiol. 33 (2), 156–161. 10.1097/ACO.0000000000000830 31904697

[B29] Deng T. Zhang Y. Yao Y. Ye P. Zhang D. Cheng F. (2025). Exosome therapeutics: a paradigm shift in skin repair through multidimensional immunomodulation and biomaterial-driven delivery. Biomed. Pharmacother. 193, 118830. 10.1016/j.biopha.2025.118830 41319522

[B30] Di Naro M. Petronio Petronio G. Mukhtar F. Cutuli M. A. Magnifico I. Falcone M. (2025). Extracellular vesicles in bacteria, archaea, and eukaryotes: mechanisms of inter-kingdom communication and clinical implications. Microorganisms 13 (3), 636. 10.3390/microorganisms13030636 40142528 PMC11944275

[B31] Donoso‐Quezada J. Ayala‐Mar S. González‐Valdez J. (2021). The role of lipids in exosome biology and intercellular communication: function, analytics and applications. Traffic 22 (7), 204–220. 10.1111/tra.12803 34053166 PMC8361711

[B32] Doyle L. M. Wang M. Z. (2019). Overview of extracellular vesicles, their origin, composition, purpose, and methods for exosome isolation and analysis. Cells 8 (7), 727. 10.3390/cells8070727 31311206 PMC6678302

[B33] Elgezawi M. Haridy R. Almas K. Abdalla M. A. Omar O. Abuohashish H. (2022). Matrix metalloproteinases in dental and periodontal tissues and their current inhibitors: developmental, degradational and pathological aspects. Int. J. Mol. Sci. 23 (16), 8929. 10.3390/ijms23168929 36012195 PMC9409155

[B34] Fafián-Labora J. Lesende-Rodriguez I. Fernández-Pernas P. Sangiao-Alvarellos S. Monserrat L. Arntz O. J. (2017). Effect of age on pro-inflammatory miRNAs contained in mesenchymal stem cell-derived extracellular vesicles. Sci. Rep. 7, 43923. 10.1038/srep43923 28262816 PMC5338265

[B35] Fang Y. Wang Z. Liu X. Tyler B. M. (2022). Biogenesis and biological functions of extracellular vesicles in cellular and organismal communication with microbes. Front. Microbiol. 13, 817844. 10.3389/fmicb.2022.817844 35250933 PMC8895202

[B36] Gao P. Li C. Chang Z. Wang X. Xuan M. (2018). Carcinoma associated fibroblasts derived from oral squamous cell carcinoma promote lymphangiogenesis via c-Met/PI3K/AKT *in vitro* . Oncol. Lett. 15 (1), 331–337. 10.3892/ol.2017.7301 29375714 PMC5766077

[B37] Gao Y. Yuan Z. Yuan X. Wan Z. Yu Y. Zhan Q. (2022). Bioinspired porous microspheres for sustained hypoxic exosomes release and vascularized bone regeneration. Bioact. Mater 14, 377–388. 10.1016/j.bioactmat.2022.01.041 35386817 PMC8964815

[B38] Ginini L. Billan S. Fridman E. Gil Z. (2022). Insight into extracellular vesicle-cell communication: from cell recognition to intracellular fate. Cells 11 (9), 1375. 10.3390/cells11091375 35563681 PMC9101098

[B39] Gregory C. D. Rimmer M. P. (2023). Extracellular vesicles arising from apoptosis: forms, functions, and applications. J. Pathol. 260 (5), 592–608. 10.1002/path.6138 37294158 PMC10952477

[B40] Guo H. Jiang W. Huang S. Huang X. Li C. (2021). Serum exosome-derived biomarkers for the early detection of oral squamous cell carcinoma. Mol. Cell Biochem. 476 (12), 4435–4447. 10.1007/s11010-021-04254-7 34468926

[B41] Gurung S. Perocheau D. Touramanidou L. Baruteau J. (2021). The exosome journey: from biogenesis to uptake and intracellular signalling. Cell Commun. Signal 19 (1), 47. 10.1186/s12964-021-00730-1 33892745 PMC8063428

[B42] Haidar Z. S. (2018). Exosomes: human saliva-derived nanoBiomarkers for use in clinical dentistry? Int. J. Odontostomatol. 12, 5–6. 10.4067/s0718-381x2018000100005

[B43] Hammouda D. A. Mansour A. M. Saeed M. A. Zaher A. R. Grawish M. E. (2023). Stem cell-derived exosomes for dentin-pulp complex regeneration: a mini-review. Restor. Dent. Endod. 48 (2), e20. 10.5395/rde.2023.48.e20 37284341 PMC10240090

[B44] Han Y. Jia L. Zheng Y. Li W. (2018). Salivary exosomes: emerging roles in systemic disease. Int. J. Biol. Sci. 14 (6), 633–643. 10.7150/ijbs.25018 29904278 PMC6001649

[B45] Hazrati A. Soudi S. Malekpour K. Mahmoudi M. Rahimi A. Hashemi S. M. (2022). Immune cells-derived exosomes function as a double-edged sword: role in disease progression and their therapeutic applications. Biomark. Res. 10 (1), 30. 10.1186/s40364-022-00374-4 35550636 PMC9102350

[B46] He L. Ping F. Fan Z. Zhang C. Deng M. Cheng B. (2020). Salivary exosomal miR-24-3p serves as a potential detective biomarker for oral squamous cell carcinoma screening. Biomed. Pharmacother. 121, 109553. 10.1016/j.biopha.2019.109553 31704611

[B47] Hessvik N. P. Øverbye A. Brech A. Torgersen M. L. Jakobsen I. S. Sandvig K. (2016). PIKfyve inhibition increases exosome release and induces secretory autophagy. Cell Mol. Life Sci. 73 (24), 4717–4737. 10.1007/s00018-016-2309-8 27438886 PMC11108566

[B48] Houschyar K. S. Tapking C. Borrelli M. R. Popp D. Duscher D. Maan Z. N. (2018). Wnt pathway in bone repair and regeneration - what do we know So far. Front. Cell Dev. Biol. 6, 170. 10.3389/fcell.2018.00170 30666305 PMC6330281

[B49] Hua S. Bartold P. M. Gulati K. Moran C. S. Ivanovski S. Han P. (2021). Periodontal and dental pulp cell-derived small extracellular vesicles: a review of the current status. Nanomaterials 11 (7), 1858. 10.3390/nano11071858 34361246 PMC8308278

[B50] Huang Y. Li R. Ye S. Lin S. Yin G. Xie Q. (2020). Recent advances in the use of exosomes in Sjögren’s syndrome. Front. Immunol. 11, 1509. 10.3389/fimmu.2020.01509 32903777 PMC7438915

[B51] Huang X. Hu X. Zhao M. Zhang Q. (2020). Analysis of salivary exosomal proteins in young adults with severe periodontitis. Oral Dis. 26 (1), 173–181. 10.1111/odi.13217 31630466

[B52] Hwang H. S. Lee C. S. (2024). Exosome-integrated hydrogels for bone tissue engineering. Gels 10 (12), 762. 10.3390/gels10120762 39727520 PMC11675329

[B53] Jiang M. Yu Y. Luo J. Gao Q. Zhang L. Wang Q. (2017). Bone marrow-derived mesenchymal stem cells expressing thioredoxin 1 attenuate bleomycin-induced skin fibrosis and oxidative stress in scleroderma. J. Invest Dermatol 137 (6), 1223–1233. 10.1016/j.jid.2017.01.011 28132855

[B54] Jing H. He X. Zheng J. (2018). Exosomes and regenerative medicine: state of the art and perspectives. Transl. Res. 196, 1–16. 10.1016/j.trsl.2018.01.005 29432720

[B55] Joshi H. J. Narimatsu Y. Schjoldager K. T. Tytgat H. L. P. Aebi M. Clausen H. (2018). SnapShot: O-glycosylation pathways across kingdoms. Cell 172 (3), 632–632.e2. 10.1016/j.cell.2018.01.016 29373833

[B56] Ju Y. Hu Y. Yang P. Xie X. Fang B. (2023). Extracellular vesicle-loaded hydrogels for tissue repair and regeneration. Mater Today Bio 18, 100522. 10.1016/j.mtbio.2022.100522 36593913 PMC9803958

[B57] Kalluri R. LeBleu V. S. (2020). The biology, function, and biomedical applications of exosomes. Science 367 (6478), eaau6977. 10.1126/science.aau6977 32029601 PMC7717626

[B58] Kim G. Kim K. H. Seo M. Kim T. Chun H. (2024). High-throughput exosome isolation based on bidirectional flow control through nanoporous membrane. Sensors Actuators B Chem. 418, 136233. 10.1016/j.snb.2024.136233

[B59] Ko S. Y. Naora H. (2020). Extracellular vesicle membrane-associated proteins: emerging roles in tumor angiogenesis and anti-angiogenesis therapy resistance. Int. J. Mol. Sci. 21 (15), 5418. 10.3390/ijms21155418 32751440 PMC7432555

[B60] Kong Q. Wang Y. Jiang N. Wang Y. Wang R. Hu X. (2024). Exosomes as promising therapeutic tools for regenerative endodontic therapy. Biomolecules 14 (3), 330. 10.3390/biom14030330 38540750 PMC10967740

[B61] Krylova S. V. Feng D. (2023). The machinery of exosomes: biogenesis, release, and uptake. Int. J. Mol. Sci. 24 (2), 1337. 10.3390/ijms24021337 36674857 PMC9865891

[B62] Lange M. Babczyk P. Tobiasch E. (2024). Exosomes: a new hope for angiogenesis-mediated bone regeneration. Int. J. Mol. Sci. 25 (10), 5204. 10.3390/ijms25105204 38791243 PMC11120942

[B63] LeBleu V. S. Kalluri R. (2020). Exosomes as a multicomponent biomarker platform in cancer. Trends Cancer 6 (9), 767–774. 10.1016/j.trecan.2020.03.007 32307267

[B64] Lee J. Y. Kim H. S. (2021). Extracellular vesicles in regenerative medicine: potentials and challenges. Tissue Eng. Regen. Med. 18 (4), 479–484. 10.1007/s13770-021-00365-w 34297340 PMC8300067

[B65] Lee J. Lee J. H. Chakraborty K. Hwang J. Lee Y. K. (2022). Exosome-based drug delivery systems and their therapeutic applications. RSC Adv. 12 (29), 18475–18492. 10.1039/d2ra02351b 35799926 PMC9218984

[B66] Li Y. J. Wu J. Y. Wang J. M. Hu X. B. Cai J. X. Xiang D. X. (2020). Gemcitabine loaded autologous exosomes for effective and safe chemotherapy of pancreatic cancer. Acta Biomater. 101, 519–530. 10.1016/j.actbio.2019.10.022 31629893

[B67] Li X. Xie X. Lian W. Shi R. Han S. Zhang H. (2018). Exosomes from adipose-derived stem cells overexpressing Nrf2 accelerate cutaneous wound healing by promoting vascularization in a diabetic foot ulcer rat model. Exp. Mol. Med. 50 (4), 1–14. 10.1038/s12276-018-0058-5 29651102 PMC5938041

[B68] Li K. Chen Y. Li A. Tan C. Liu X. (2019). Exosomes play roles in sequential processes of tumor metastasis. Int. J. Cancer 144 (7), 1486–1495. 10.1002/ijc.31774 30155891

[B69] Li Q. Yu H. Sun M. Yang P. Hu X. Ao Y. (2021). The tissue origin effect of extracellular vesicles on cartilage and bone regeneration. Acta Biomater. 125, 253–266. 10.1016/j.actbio.2021.02.039 33657452

[B70] Li M. Fang F. Sun M. Zhang Y. Hu M. Zhang J. (2022). Extracellular vesicles as bioactive nanotherapeutics: an emerging paradigm for regenerative medicine. Theranostics 12 (11), 4879–4903. 10.7150/thno.72812 35836815 PMC9274746

[B71] Li K. L. Huang H. Y. Ren H. Yang X. L. (2022). Role of exosomes in the pathogenesis of inflammation in Parkinson’s disease. Neural Regen. Res. 17 (9), 1898–1906. 10.4103/1673-5374.335143 35142665 PMC8848593

[B72] Li B. Xian X. Lin X. Huang L. Liang A. Jiang H. (2022). Hypoxia alters the proteome profile and enhances the angiogenic potential of dental pulp stem cell-derived exosomes. Biomolecules 12 (4), 575. 10.3390/biom12040575 35454164 PMC9029684

[B73] Li N. Wang J. Feng G. Liu Y. Shi Y. Wang Y. (2024). Advances in biomaterials for oral-maxillofacial bone regeneration: spotlight on periodontal and alveolar bone strategies. Regen. Biomater. 11, rbae078. 10.1093/rb/rbae078 39055303 PMC11272181

[B74] Liao J. Wu T. Zhang Q. Shen P. Huang Z. Wang J. (2026). TGF-β/BMP signaling in skeletal biology: molecular mechanisms, regulatory networks, and therapeutic implications in development, regeneration, and disease. Bone Res. 14 (1), 6. 10.1038/s41413-025-00497-y 41530114 PMC12800155

[B75] Lin Y. Anderson J. D. Rahnama L. M. A. Gu S. V. Knowlton A. A. (2020). Exosomes in disease and regeneration: biological functions, diagnostics, and beneficial effects. Am. J. Physiol. Heart Circ. Physiol. 319 (6), H1162–h1180. 10.1152/ajpheart.00075.2020 32986962 PMC7792703

[B76] Liu Z. Wang S. Huo N. Yang S. Shi Q. Xu J. (2022). Extracellular vesicles: a potential future strategy for dental and maxillofacial tissue repair and regeneration. Front. Physiol. 13, 1012241. 10.3389/fphys.2022.1012241 36479350 PMC9719951

[B77] Lorite P. Domínguez J. N. Palomeque T. Torres M. I. (2024). Extracellular vesicles: advanced tools for disease diagnosis, monitoring, and therapies. Int. J. Mol. Sci. 26 (1), 189. 10.3390/ijms26010189 39796048 PMC11720073

[B78] Lu C. L. (2015). An efficient algorithm for the contig ordering problem under algebraic rearrangement distance. J. Comput. Biol. 22 (11), 975–987. 10.1089/cmb.2015.0073 26247343

[B79] Macia L. Nanan R. Hosseini-Beheshti E. Grau G. E. (2019). Host- and microbiota-derived extracellular vesicles, immune function, and disease development. Int. J. Mol. Sci. 21 (1), 107. 10.3390/ijms21010107 31877909 PMC6982009

[B80] Malla R. R. Pandrangi S. Kumari S. Gavara M. M. Badana A. K. (2018). Exosomal tetraspanins as regulators of cancer progression and metastasis and novel diagnostic markers. Asia Pac J. Clin. Oncol. 14 (6), 383–391. 10.1111/ajco.12869 29575602

[B81] Mao Y. Wang Y. Dong L. Zhang Y. Zhang Y. Wang C. (2019). Hypoxic exosomes facilitate angiogenesis and metastasis in esophageal squamous cell carcinoma through altering the phenotype and transcriptome of endothelial cells. J. Exp. Clin. Cancer Res. 38 (1), 389. 10.1186/s13046-019-1384-8 31488217 PMC6727585

[B82] Mathieu M. Martin-Jaular L. Lavieu G. Théry C. (2019). Specificities of secretion and uptake of exosomes and other extracellular vesicles for cell-to-cell communication. Nat. Cell Biol. 21 (1), 9–17. 10.1038/s41556-018-0250-9 30602770

[B83] Matsui M. Tabata Y. (2012). Enhanced angiogenesis by multiple release of platelet-rich plasma contents and basic fibroblast growth factor from gelatin hydrogels. Acta Biomater. 8 (5), 1792–1801. 10.1016/j.actbio.2012.01.016 22293581

[B84] Mattei V. Delle Monache S. (2023). Dental pulp stem cells (DPSCs) and tissue regeneration: mechanisms mediated by direct, paracrine, or autocrine effects. Biomedicines 11 (2), 386. 10.3390/biomedicines11020386 36830923 PMC9953448

[B85] Miao Q. Li S. Lyu W. Zhang J. Han Y. (2025). Exosomes in oral diseases: mechanisms and therapeutic applications. Drug Des. Devel Ther. 19, 457–469. 10.2147/dddt.s505355 PMC11766710 39867866

[B86] Moreno C. M. Boeree E. Freitas C. M. T. Weber K. S. (2023). Immunomodulatory role of oral microbiota in inflammatory diseases and allergic conditions. Front. Allergy 4, 1067483. 10.3389/falgy.2023.1067483 36873050 PMC9981797

[B87] Mottaghi M. Kianbakht C. Samadian S. Farshbaf A. Mirhashemi M. Ahrari F. (2025). Clinical applications of dental pulp stem cells-derived exosomes in oral and maxillofacial tissue regeneration and repair. Differentiation 146, 100918. 10.1016/j.diff.2025.100918 41308294

[B88] Nakamichi E. Sakakura H. Mii S. Yamamoto N. Hibi H. Asai M. (2021). Detection of serum/salivary exosomal Alix in patients with oral squamous cell carcinoma. Oral Dis. 27 (3), 439–447. 10.1111/odi.13565 32688445

[B89] Ning X. Liu R. Huang Y. Huang Z. Li H. Li Q. (2024). Dental stem cell-derived exosomes: a review of their isolation, classification, functions, and mechanisms. Stem Cells Int. 2024, 2187392. 10.1155/2024/2187392 39184549 PMC11343633

[B90] Norouzi F. Aghajani S. Vosoughi N. Sharif S. Ghahremanzadeh K. Mokhtari Z. (2024). Exosomes derived stem cells as a modern therapeutic approach for skin rejuvenation and hair regrowth. Regen. Ther. 26, 1124–1137. 10.1016/j.reth.2024.10.001 39640923 PMC11617408

[B91] Palazon A. Goldrath A. W. Nizet V. Johnson R. S. (2014). HIF transcription factors, inflammation, and immunity. Immunity 41 (4), 518–528. 10.1016/j.immuni.2014.09.008 25367569 PMC4346319

[B92] Panfoli I. Bruschi M. (2020). The good and bad sides of exosomes: pre-metastatic niche formation, cancer biomarker and therapy carriers. J. Cancer Metastasis Treat. 6, 35–42. 10.20517/2394-4722.2020.50

[B93] Paskeh M. D. A. Entezari M. Mirzaei S. Zabolian A. Saleki H. Naghdi M. J. (2022). Emerging role of exosomes in cancer progression and tumor microenvironment remodeling. J. Hematol. Oncol. 15 (1), 83. 10.1186/s13045-022-01305-4 35765040 PMC9238168

[B94] Patel G. K. Khan M. A. Zubair H. Srivastava S. K. Khushman M. Singh S. (2019). Comparative analysis of exosome isolation methods using culture supernatant for optimum yield, purity and downstream applications. Sci. Rep. 9 (1), 5335. 10.1038/s41598-019-41800-2 30926864 PMC6441044

[B95] Peng S. Cao L. He S. Zhong Y. Ma H. Zhang Y. (2018). An overview of long noncoding RNAs involved in bone regeneration from mesenchymal stem cells. Stem Cells Int. 2018, 8273648. 10.1155/2018/8273648 29535782 PMC5829309

[B96] Peng Q. Zhang J. Zhou G. (2018). Differentially circulating exosomal microRNAs expression profiling in oral lichen planus. Am. J. Transl. Res. 10 (9), 2848–2858. 30323871 PMC6176222

[B97] Petroni D. Fabbri C. Babboni S. Menichetti L. Basta G. Del Turco S. (2023). Extracellular vesicles and intercellular communication: challenges for *in vivo* molecular imaging and tracking. Pharmaceutics 15 (6), 1639. 10.3390/pharmaceutics15061639 37376087 PMC10301899

[B98] Qoronfleh M. W. Al-Dewik N. (2025). Cancer biomarkers: reflection on recent progress, emerging innovations, and the clinical horizon. Cancers 17 (18), 2981. 10.3390/cancers17182981 41008828 PMC12468363

[B99] Ráez-Meseguer C. Navas-Enamorado C. Capó X. Galmes-Panades A. Molina de la Llave A. Mendez-Varela M. (2026). MicroRNA profiles in plasma-derived extracellular vesicles across the human lifespan. NPJ Aging 12 (1), 30. 10.1038/s41514-025-00321-1 41690959 PMC12909829

[B100] Rajput A. Varshney A. Bajaj R. Pokharkar V. (2022). Exosomes as new generation vehicles for drug delivery: biomedical applications and future perspectives. Molecules 27 (21), 7289. 10.3390/molecules27217289 36364116 PMC9658823

[B101] Raposo G. Stoorvogel W. (2013). Extracellular vesicles: exosomes, microvesicles, and friends. J. Cell Biol. 200 (4), 373–383. 10.1083/jcb.201211138 23420871 PMC3575529

[B102] Riau A. K. Ong H. S. Yam G. H. F. Mehta J. S. (2019). Sustained delivery system for stem cell-derived exosomes. Front. Pharmacol. 10, 1368. 10.3389/fphar.2019.01368 31798457 PMC6868085

[B103] Salomon C. Das S. Erdbrügger U. Kalluri R. Kiang Lim S. Olefsky J. M. (2022). Extracellular vesicles and their emerging roles as cellular messengers in endocrinology: an endocrine society scientific statement. Endocr. Rev. 43 (3), 441–468. 10.1210/endrev/bnac009 35552682 PMC10686249

[B104] Sanesi L. Mori G. Troiano G. Ballini A. Valzano F. Dioguardi M. (2024). Salivary exosomal microRNA profile as biomonitoring tool for diagnosis and prognosis of patients with head and neck squamous cell carcinoma: a systematic review. Arch. Oral Biol. 165, 106012. 10.1016/j.archoralbio.2024.106012 38879952

[B105] Santavanond J. P. Rutter S. F. Atkin-Smith G. K. Poon I. K. H. (2021). Apoptotic bodies: mechanism of formation, isolation and functional relevance. Subcell. Biochem. 97, 61–88. 10.1007/978-3-030-67171-6_4 33779914

[B106] Schank M. B. Zhao J. Wang L. Moorman J. P. Yao Z. Q. (2026). Extracellular vesicles: a comprehensive review of their origins, functions, and therapeutic potential. Biomedicines 14, 495. 10.3390/biomedicines14030495 41898142 PMC13023759

[B107] Shetty S. S. Sowmya S. Pradeep A. Jayakumar R. (2025). Gingival mesenchymal stem cells: a periodontal regenerative substitute. Tissue Eng. Regen. Med. 22 (1), 1–21. 10.1007/s13770-024-00676-8 39607668 PMC11711796

[B108] Shimizu Y. Takeda‐Kawaguchi T. Kuroda I. Hotta Y. Kawasaki H. Hariyama T. (2022). Exosomes from dental pulp cells attenuate bone loss in mouse experimental periodontitis. J. Periodontal Res. 57 (1), 162–172. 10.1111/jre.12949 34826339

[B109] Skallevold H. E. Rokaya D. Khurshid Z. Zafar M. S. (2019). Bioactive glass applications in dentistry. Int. J. Mol. Sci. 20 (23), 5960. 10.3390/ijms20235960 31783484 PMC6928922

[B110] Ståhl A. L. Johansson K. Mossberg M. Kahn R. Karpman D. (2019). Exosomes and microvesicles in normal physiology, pathophysiology, and renal diseases. Pediatr. Nephrol. 34 (1), 11–30. 10.1007/s00467-017-3816-z 29181712 PMC6244861

[B111] Stanko P. Altanerova U. Jakubechova J. Repiska V. Altaner C. (2018). Dental mesenchymal stem/stromal cells and their exosomes. Stem Cells Int. 2018 (1), 8973613. 10.1155/2018/8973613 29760738 PMC5924966

[B112] Sun D. S. Chang H. H. (2024a). Extracellular vesicles: function, resilience, biomarker, bioengineering, and clinical implications. Tzu Chi Med. J. 36 (3), 251–259. 10.4103/tcmj.tcmj_28_24 38993825 PMC11236075

[B113] Sun M. Yu Y. Zhang W. Ding Y. Li A. Li Y. (2024b). Extracellular vesicles derived from dental follicle stem cells regulate tooth eruption by inhibiting osteoclast differentiation. Front. Cell Dev. Biol. 12, 1503481. 10.3389/fcell.2024.1503481 39834384 PMC11744031

[B114] Tawanwongsri W. Vachiramon V. (2024). Skin necrosis after intradermal injection of lyophilized exosome: a case report and a review of the literature. J. Cosmet. Dermatol 23 (5), 1597–1603. 10.1111/jocd.16206 38327119

[B115] Thakur A. Rai D. (2024). Global requirements for manufacturing and validation of clinical grade extracellular vesicles. J. Liq. Biopsy 6, 100278. 10.1016/j.jlb.2024.100278 40027307 PMC11863704

[B116] Thakur A. Shah D. Rai D. Parra D. C. Pathikonda S. Kurilova S. (2023). Therapeutic values of exosomes in cosmetics, skin care, tissue regeneration, and dermatological diseases. Cosmetics 10 (2), 65. 10.3390/cosmetics10020065

[B117] Tobón-Arroyave S. I. Celis-Mejía N. Córdoba-Hidalgo M. P. Isaza-Guzmán D. M. (2019). Decreased salivary concentration of CD9 and CD81 exosome-related tetraspanins may be associated with the periodontal clinical status. J. Clin. Periodontol. 46 (4), 470–480. 10.1111/jcpe.13099 30825338

[B118] Vafaei S. Mansoori M. Hashemi F. Basiri M. (2022). Exosome odyssey to original line in dental regeneration. J. Oral Biosci. 64 (3), 271–278. 10.1016/j.job.2022.05.002 35589074

[B119] van Niel G. D’Angelo G. Raposo G. (2018). Shedding light on the cell biology of extracellular vesicles. Nat. Rev. Mol. Cell Biol. 19 (4), 213–228. 10.1038/nrm.2017.125 29339798

[B120] Wang D. R. Pan J. (2023). Extracellular vesicles: emerged as a promising strategy for regenerative medicine. World J. Stem Cells 15 (4), 165–181. 10.4252/wjsc.v15.i4.165 37181006 PMC10173817

[B121] Wang Z. Maruyama K. Sakisaka Y. Suzuki S. Tada H. Suto M. (2019). Cyclic stretch force induces periodontal ligament cells to secrete exosomes that suppress IL-1β production through the inhibition of the NF-κB signaling pathway in macrophages. Front. Immunol. 10, 1310. 10.3389/fimmu.2019.01310 31281309 PMC6595474

[B122] Wang W. Xu Z. Liu M. Cai M. Liu X. (2023). Prospective applications of extracellular vesicle-based therapies in regenerative medicine: implications for the use of dental stem cell-derived extracellular vesicles. Front. Bioeng. Biotechnol. 11, 1278124. 10.3389/fbioe.2023.1278124 37936823 PMC10627172

[B123] Wang J. Jing J. Zhou C. Fan Y. (2024). Emerging roles of exosomes in oral diseases progression. Int. J. Oral Sci. 16 (1), 4. 10.1038/s41368-023-00274-9 38221571 PMC10788352

[B124] Wang C. K. Tsai T. H. Lee C. H. (2024). Regulation of exosomes as biologic medicines: regulatory challenges faced in exosome development and manufacturing processes. Clin. Transl. Sci. 17 (8), e13904. 10.1111/cts.13904 39115257 PMC11307316

[B125] Wang B. Lyu F. J. Deng Z. Zheng Q. Ma Y. Peng Y. (2025). Therapeutic potential of stem cell-derived exosomes for bone tissue regeneration around prostheses. J. Orthop. Transl. 52, 85–96. 10.1016/j.jot.2025.03.007 PMC12023751 40291635

[B126] Wang Y. Mao J. Wang Y. Wang R. Jiang N. Hu X. (2025). Odontogenic exosomes simulating the developmental microenvironment promote complete regeneration of pulp-dentin complex *in vivo* . J. Adv. Res. 76, 405–421. 10.1016/j.jare.2024.12.048 39765328 PMC12793753

[B127] Welsh J. A. Goberdhan D. C. I. O'Driscoll L. Buzas E. I. Blenkiron C. Bussolati B. (2024). Minimal information for studies of extracellular vesicles (MISEV2023): from basic to advanced approaches. J. Extracell. Vesicles 13 (2), e12404. 10.1002/jev2.12404 38326288 PMC10850029

[B128] Wollert T. Hurley J. H. (2010). Molecular mechanism of multivesicular body biogenesis by ESCRT complexes. Nature 464 (7290), 864–869. 10.1038/nature08849 20305637 PMC2851844

[B129] Wronowska E. Guevara-Lora I. Brankiewicz A. Bras G. Zawrotniak M. Satala D. (2025). Synergistic effects of Candida albicans and Porphyromonas gingivalis biofilms on epithelial barrier function in a 3D aspiration pneumonia model. Front. Cell Infect. Microbiol. 15, 1552395. 10.3389/fcimb.2025.1552395 40125517 PMC11925950

[B130] Wu J. Liu G. Jia R. Guo J. (2023). Salivary extracellular vesicles: biomarkers and beyond in human diseases. Int. J. Mol. Sci. 24 (24), 17328. 10.3390/ijms242417328 38139157 PMC10743646

[B131] Wu Z. Long W. Yin Y. Tan B. Liu C. Li H. (2025). Outer membrane vesicles of porphyromonas gingivalis: recent advances in pathogenicity and associated mechanisms. Front. Microbiol. 16, 1555868. 10.3389/fmicb.2025.1555868 40256625 PMC12007433

[B132] Xia Y. He X. T. Xu X. Y. Tian B. M. An Y. Chen F. M. (2020). Exosomes derived from M0, M1 and M2 macrophages exert distinct influences on the proliferation and differentiation of mesenchymal stem cells. PeerJ 8, e8970. 10.7717/peerj.8970 32355576 PMC7185029

[B133] Xia E. J. Zou S. Zhao X. Liu W. Zhang Y. Zhao I. S. (2024). Extracellular vesicles as therapeutic tools in regenerative dentistry. Stem Cell Res. Ther. 15 (1), 365. 10.1186/s13287-024-03936-5 39402576 PMC11476107

[B134] Xie L. Wang J. Zhang Y. Chen H. Lin D. Ding J. (2019). The effects of local injection of exosomes derived from BMSCs on random skin flap in rats. Am. J. Transl. Res. 11 (11), 7063–7073. 31814909 PMC6895502

[B135] Xing X. Han S. Li Z. Li Z. (2020). Emerging role of exosomes in craniofacial and dental applications. Theranostics 10 (19), 8648–8664. 10.7150/thno.48291 32754269 PMC7392016

[B136] Xu R. Greening D. W. Zhu H. J. Takahashi N. Simpson R. J. (2016). Extracellular vesicle isolation and characterization: toward clinical application. J. Clin. Invest 126 (4), 1152–1162. 10.1172/JCI81129 27035807 PMC4811150

[B137] Xu M. Ji J. Jin D. Wu Y. Wu T. Lin R. (2023). The biogenesis and secretion of exosomes and multivesicular bodies (MVBs): intercellular shuttles and implications in human diseases. Genes Dis. 10 (5), 1894–1907. 10.1016/j.gendis.2022.03.021 37492712 PMC10363595

[B138] Yáñez-Mó M. Siljander P. R. M. Andreu Z. Zavec A. B. Borràs F. E. Buzas E. I. (2015). Biological properties of extracellular vesicles and their physiological functions. J. Extracell. Vesicles 4, 27066. 10.3402/jev.v4.27066 25979354 PMC4433489

[B139] Yang S. J. Wang D. D. Li J. Xu H. Z. Shen H. Y. Chen X. (2017). Predictive role of GSTP1-containing exosomes in chemotherapy-resistant breast cancer. Gene 623, 5–14. 10.1016/j.gene.2017.04.031 28438694

[B140] Yang Y. N. Zhang R. Du J. W. Yuan H. H. Li Y. J. Wei X. L. (2018). Predictive role of UCA1-containing exosomes in cetuximab-resistant colorectal cancer. Cancer Cell Int. 18, 164. 10.1186/s12935-018-0660-6 30377411 PMC6196422

[B141] Yang C. Zhang M. Sung J. Wang L. Jung Y. Merlin D. (2020). Autologous exosome transfer: a new personalised treatment concept to prevent colitis in a murine model. J. Crohns Colitis 14 (6), 841–855. 10.1093/ecco-jcc/jjz184 31710674 PMC7346889

[B142] Yang Y. Zhang B. Yang Y. Peng B. Ye R. (2022). PLGA containing human adipose-derived stem cell-derived extracellular vesicles accelerates the repair of alveolar bone defects via transfer of CGRP. Oxid. Med. Cell Longev. 2022, 4815284. 10.1155/2022/4815284 35726333 PMC9206573

[B143] Yasothkumar D. Ramani P. Jayaraman S. Ramalingam K. Tilakaratne W. M. (2024). Expression profile of circulating exosomal microRNAs in leukoplakia, oral submucous fibrosis, and combined lesions of leukoplakia and oral submucous fibrosis. Head. Neck Pathol. 18 (1), 28. 10.1007/s12105-024-01627-4 38536520 PMC10973321

[B144] Yong T. Zhang X. Bie N. Zhang H. Zhang X. Li F. (2019). Tumor exosome-based nanoparticles are efficient drug carriers for chemotherapy. Nat. Commun. 10 (1), 3838. 10.1038/s41467-019-11718-4 31444335 PMC6707218

[B145] Yu M. Y. Liu X. C. Yu Z. L. Jia J. (2024). The application of salivary exosomes in the diagnosis of oral disease. Chin. J. Dent. Res. 27 (4), 291–301. 10.3290/j.cjdr.b5860259 39641292

[B146] Yu C. Zheng D. Xu C. Wang T. Xu J. (2024). Global research trends of nanomaterials application in periodontitis and peri-implantitis: a bibliometric analysis. Heliyon 10 (16), e36187. 10.1016/j.heliyon.2024.e36187 39224328 PMC11367449

[B147] Zeng M. Liu M. Tao X. Yin X. Shen C. Wang X. (2024). Emerging trends in the application of extracellular vesicles as novel oral delivery vehicles for therapeutics in inflammatory diseases. Int. J. Nanomedicine 19, 8573–8601. 10.2147/IJN.S475532 39185348 PMC11345024

[B148] Zhang H. Z. Wang S. Y. (2019). Myoepithelioma-like tumour of the vulvar region. Pathology 51 (6), 665–668. 10.1016/j.pathol.2019.06.004 31470996

[B149] Zhang H. Zhuang X. Sun D. Liu Y. Xiang X. Grizzle W. E. (2012). Exosomes and immune surveillance of neoplastic lesions: a review. Biotech. Histochem 87 (3), 161–168. 10.3109/10520291003659042 22216980 PMC3445025

[B150] Zhang J. Liu X. Li H. Chen C. Hu B. Niu X. (2016). Exosomes/tricalcium phosphate combination scaffolds can enhance bone regeneration by activating the PI3K/Akt signaling pathway. Stem Cell Res. Ther. 7 (1), 136. 10.1186/s13287-016-0391-3 27650895 PMC5028974

[B151] Zhang C. Song J. Lou L. Qi X. Zhao L. Fan B. (2021). Doxorubicin-loaded nanoparticle coated with endothelial cells-derived exosomes for immunogenic chemotherapy of glioblastoma. Bioeng. Transl. Med. 6 (3), e10203. 10.1002/btm2.10203 34589592 PMC8459598

[B152] Zhang Y. Liu J. Liu S. Yu L. Liu S. Li M. (2023). Extracellular vesicles in oral squamous cell carcinoma: current progress and future prospect. Front. Bioeng. Biotechnol. 11, 1149662. 10.3389/fbioe.2023.1149662 37304135 PMC10250623

[B153] Zhang Y. Wu D. Zhou C. Bai M. Wan Y. Zheng Q. (2024). Engineered extracellular vesicles for tissue repair and regeneration. Burns Trauma 12, tkae062. 10.1093/burnst/tkae062 39439545 PMC11495891

[B154] Zhao J. Ding Y. He R. Huang K. Liu L. Jiang C. (2020). Dose-effect relationship and molecular mechanism by which BMSC-derived exosomes promote peripheral nerve regeneration after crush injury. Stem Cell Res. Ther. 11 (1), 360. 10.1186/s13287-020-01872-8 32811548 PMC7437056

[B155] Zhou T. Pan J. Wu P. Huang R. Du W. Zhou Y. (2019). Dental follicle cells: roles in development and beyond. Stem Cells Int. 2019, 9159605. 10.1155/2019/9159605 31636679 PMC6766151

[B156] Zlotogorski-Hurvitz A. Dayan D. Chaushu G. Salo T. Vered M. (2016). Morphological and molecular features of oral fluid-derived exosomes: oral cancer patients *versus* healthy individuals. J. Cancer Res. Clin. Oncol. 142 (1), 101–110. 10.1007/s00432-015-2005-3 26115960 PMC11819233

[B157] Zou J. Xia H. Jiang Q. Su Z. Wen S. Liang Z. (2023). Exosomes derived from odontogenic stem cells: its role in the dentin-pulp complex. Regen. Ther. 24, 135–146. 10.1016/j.reth.2023.05.008 37415682 PMC10320411

